# Hypoxia in extravillous trophoblasts links maternal obesity and offspring neurobehavior

**DOI:** 10.1016/j.isci.2025.112636

**Published:** 2025-05-12

**Authors:** Fatima Gunter-Rahman, Shayna Mallett, Frédérique White, Pierre-Étienne Jacques, Ravikiran M. Raju, Marie-France Hivert, Eunjung Alice Lee

**Affiliations:** 1 Harvard-MIT Program in Health Sciences and Technology, Massachusetts Institute of Technology, Cambridge, MA, USA; 2 Division of Genetics and Genomics, Boston Children’s Hospital, Boston, MA, USA; 3 Department of Pediatrics, Harvard Medical School, Boston, MA, USA; 4 Département de Biologie, Faculté des Sciences, Université de Sherbrooke, Sherbrooke, QC, Canada; 5 Centre de Recherche du Centre Hospitalier Universitaire de Sherbrooke, Sherbrooke, QC, Canada; 6 Institut de Recherche sur le Cancer de l’Université de Sherbrooke, Sherbrooke, QC, Canada; 7 Division of Newborn Medicine, Boston Children’s Hospital, Harvard Medical School, Boston, MA, USA; 8 Picower Institute for Learning and Memory, Massachusetts Institute of Technology, Cambridge, MA, USA; 9 Department of Population Medicine, Harvard Pilgrim Health Care Institute and Harvard Medical School, Boston, MA, USA; 10 Diabetes Unit, Endocrine Division, Department of Medicine, Massachusetts General Hospital, Boston, MA, USA; 11 Broad Institute of MIT and Harvard, Cambridge, MA 02142, USA

**Keywords:** Molecular biology, Neuroscience

## Abstract

While maternal obesity (MO) is associated with neurobehavioral impairment (NBI) in offspring, the underlying mechanisms remain unknown. The placenta is thought to play a role in fetal programming. To elucidate the association between MO and offspring NBI, we performed single-nucleus RNA-seq on maternal- and fetal-facing sides of human term placentas from MO and lean groups. MO placentas showed the upregulation of hypoxia response genes in multiple cell types, and maternal-facing hypoxia gene expression correlated with offspring NBI in an independent birth cohort, Gen3G. Extravillous trophoblasts (EVTs) showed the highest expression of NBI-correlated genes, and EVT NBI-gene expression correlated with hypoxia signatures in two cohorts. Exposing cultured EVTs to hypoxia increased NBI gene expression, and 44% of the association between maternal BMI and NBI-gene expression in EVTs was mediated by hypoxia. Our findings suggest that hypoxia in EVTs is a key process in the neurodevelopmental programming of fetal exposure to MO.

## Highlights

• Placental cells of fetal origin show a hypoxic response to maternal obesity
• Hypoxia gene scores in placenta RNAseq correlate with biological signals of hypoxia
• Maternal side placental hypoxia correlates with abnormal neurobehavioral testing
• Extravillous trophoblasts are a key link between maternal obesity and neurobehavior

## Graphical abstract

**Figure:**
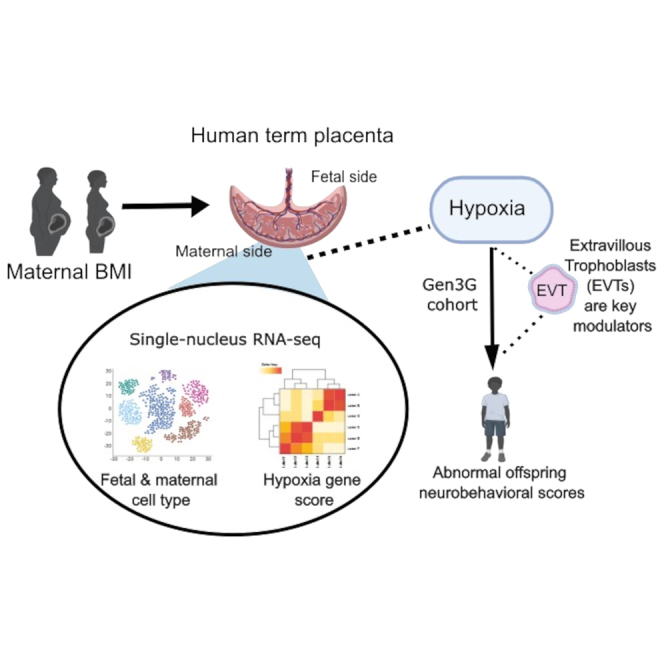

## Introduction

The prevalence of obesity, both during pregnancy and generally in the population, is increasing.^1^ 29% of women in America enter pregnancy with obesity, defined as a body mass index (BMI) ≥ 30 kg/m^2^,^2^ and the prevalence of maternal obesity (MO) is expected to go up to 47% by 2030.^3^ Similar trends are present in countries around the world.^4^ Prenatal exposure to MO is associated with life-long offspring mortality^5^ and morbidity, including cardiovascular disease, diabetes, cancer, psychiatric disorders,^1^ and obesity.^6^ Studies show that MO is associated with offspring DNA methylation patterns at birth,^7^ and later in childhood and adolescence,^8^ suggesting that epigenetic programming plays a role in the lasting effects of MO.

Understanding the placental alterations related to MO can provide insights into the developmental programming of chronic disease, potentially serving as a basis for interventions. The placenta consumes 40–60% of oxygen and glucose delivered to the fetus, despite making up only 10–20% of uterine mass.^9^ There is growing interest in the role of the placenta on neurodevelopment and mental health of the offspring later in life,^10^^,^^11^^,^^12^^,^^13^ especially since placental gene expression and DNA methylation has been associated with the offspring development of autism and schizophrenia^14^^,^^15^ later in life.

Until now, no cell-type specific profiling of the effects of MO on human placenta and trophoblasts has taken place. Previous studies have primarily examined changes in bulk gene expression,^16^^,^^17^^,^^18^^,^^19^ and have not clarified a consistent placental face for the sample site of origin. Gene expression within the same cell type differs by location within the placenta.^20^^,^^21^ Single-cell RNA seq (scRNA-seq) or single-nucleus RNA-seq (snRNA-seq) can offer insights that are missed by bulk, and snRNA-seq in particular can offer insight into syncytiotrophoblasts (SCTs),^22^^,^^23^ since their multinucleated nature leads to poor recovery from scRNA-seq.^24^^,^^25^ To our knowledge, there is only one study that examined cell-type specific transcriptomic changes associated with maternal BMI in humans, but it was limited to maternal immune cell types in the decidua,^26^ which misses some of the key cell types of interest in offspring development—including fetal macrophages (FM)^13^^,^^27^ and trophoblasts.^28^^,^^29^

Here, we elucidate cell-type specific transcriptomic changes associated with MO by generating snRNA-seq data from term placenta tissues of humans with a pre-pregnancy BMI of ≥35 kg/m^2^ (MO condition) or 18.5–25 kg/m^2^ (lean condition). We profile two spatial locations within the placenta separately: the maternal facing side and fetal facing side. Each location contains cells of both maternal and fetal origin.^20^ The maternal-facing side includes both decidual tissue and villous tissue close to the decidua, and the fetal-facing side is villous tissue. We also rely on side-specific placenta RNA-seq data (from bulk tissue) and offspring follow-up measures in childhood available from the Genetics of Glucose regulation in Gestation and Growth (Gen3G) prospective cohort to understand how placental transcriptomic changes associated with MO relate to post-natal growth and neurodevelopment.^30^

## Results

### Single nuclei profiling of the term placenta from MO and control pregnancies

We selected and received frozen term human placenta samples from the Women and Infants Health Specimen Consortium at the Washington School of Medicine in St. Louis, Missouri. We profiled nuclei from the maternal-facing side (3 lean and 7 MO samples) and fetal-facing side (4 lean and 6 MO samples) separately. All samples came from singleton pregnancies without pre-eclampsia or gestational diabetes (see STAR Methods for full exclusion criteria). Samples in each condition and each side came from both fetal sexes. Maternal age ranged from 19 to 30, and did not differ significantly between the two groups on each side (maternal side Student’s t test *p* = 0.80, fetal side Student’s t test *p* = 0.39). Additional demographic characteristics are in Table S1.

**Figure 1:**
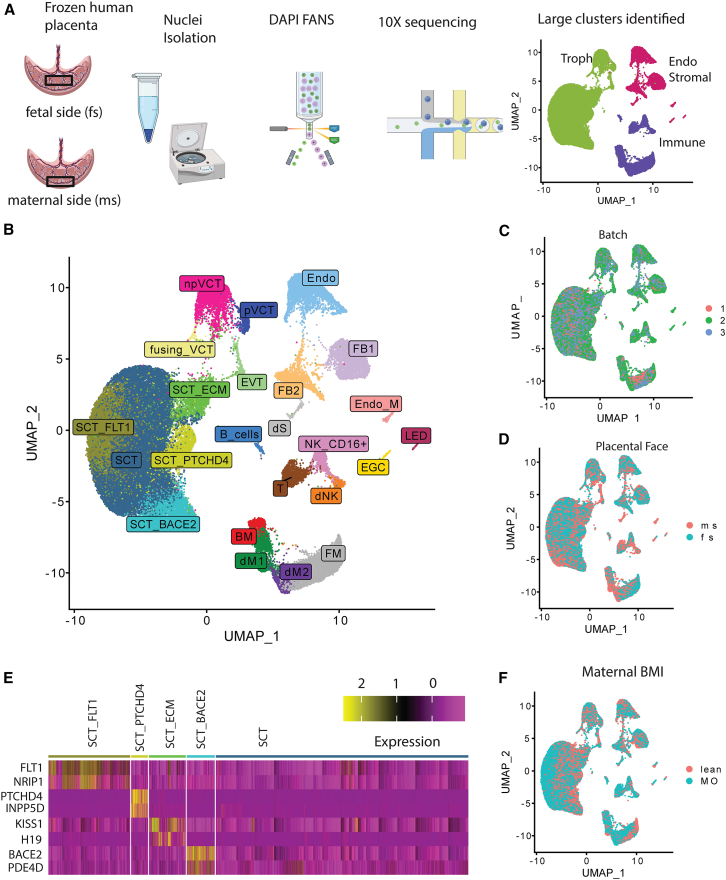
Overview of single-nucleus RNA-seq from human term placenta (A) Experimental procedures to generate single-nucleus data. (B) UMAP plot of cell types identified. (C) Batches are well integrated per UMAP colored by sequencing batch and (D) most cell types have representation on both sides of the placenta. (E) Marker gene expression of SCT subclusters identified. (F) All cell types are present in both BMI conditions. Acronyms: SCT = Syncytiotrophoblast, ECM = Extracellular matrix, BACE2, FLT1, and PTCHD4 are marker genes; VCT = villous cytotrophoblast, (n)p=(non)-proliferative, EVT = extravillous trophoblasts; FB = fibroblast, Endo = endothelial, Endo_M = maternal origin endothelial cells; LED = lymphatic endothelial cell; EGC = epithelial glandular cell; NK = natural killer; T = T cells; “d” prefix = decidual; M = macrophage; BM = Blood-derived macrophage; FM = fetal macrophage; S = stromal cell; m s = maternal side; f s = fetal side.

After filtering for low-quality nuclei (see STAR Methods, Figure 1A) we recovered 62,864 nuclei. All major cell types were well-represented: trophoblasts, immune cells, and endothelial/stromal cell populations, and sub-clustering was performed to identify detailed cell types (Figures 1A, 1B, and 1E). Batches were well integrated (Figures 1C and S1), and placental cell types were represented on both sides, other than cell types known to reside in the decidua (Figures 1D and S2). We performed a sub-analysis on male fetuses to identify the origin (fetal or maternal) of cells found in the placenta. XIST is expressed only in females, and Y chromosome genes such as USP9Y are only expressed in males. Using these marker genes, we determined that immune cells, other than fetal macrophages (FM), were of maternal origin, trophoblasts were of fetal origin, and endothelial and stromal cells were mixed (Figure S3) as expected.^20^^,^^31^ FM were further confirmed by the marker FOLR2 (Figure S4), established by Thomas et al.^32^ We further investigated monocyte and macrophage subtypes based on previously identified markers of placenta associated maternal macrophages/monocytes (PAMMs).^32^ Both dM1 and dM2 were HLA-DR+ (Figure S4), which is consistent with the expression of PAMMs. The cluster we annotated as blood-derived macrophages (BM) were also HLA-DR+ and had a higher expression of CCR2 (Figure S4), which is consistent with the expression of maternal monocytes.

As our study used snRNA-seq rather than scRNA seq, we recovered more SCTs than previous human placenta studies.^24^^,^^31^^,^^33^^,^^34^ Recently, Wang et al.^22^ identified several sub-clusters in SCTs based on early pregnancy and term multi-omics. Here, we corroborate some of their findings by performing *de novo* clustering and identifying sub-clusters. We compared our findings to the third trimester (“late”) syncytiotrophoblasts termed lSTB from Wang et al. We found clusters enriched for the expression of FLT1, which was consistent with “lSTB mature 2-a”, one of the sub-clusters in the lineage enriched for carboxylic acid transport and oxygen sensing. Another subcluster was enriched for the marker BACE2, which matched “lSTB mature 1-c”, and PTCHD4 (“lSTB nascent” predicted using trajectory analysis to be the first SCT sub-type after fusion). Our findings support that these are true sub-clusters of SCTs present in the human term placenta.

In addition, we identified another cluster (“SCT_ECM”) whose marker genes were enriched for extracellular matrix remodeling (Figure S5), with genes such as KISS1 and H19 (Figure 1E). Although this cluster expressed genes that did not match any of the markers identified in prior human placenta datasets, snRNA-seq of mouse placenta identified an SCT sub-cluster (“SynTII”) enriched for cell–matrix interactions.^35^ In addition, SCT_ECM’s meaningful functional enrichment for a key process of SCTs suggests it may play a unique role. KISS1 is synthesized by SCTs, and interacts with the KISS1-receptor on extravilllous trophoblasts (EVTs) to influence their invasiveness.^36^ Elevated KISS1 expression has been found in the placenta of women with preeclampsia.^37^ H19, a paternally imprinted gene (thus only the maternal copy is expressed),^38^ is expressed by all trophoblast cell types, and also thought to play a role in EVTs’ angiogenic capacity.^39^

Each cell type and subtype identified was present in both MO and control pregnancies (Figures S6 and S7). We did not find a significant association between MO and cell type proportion (using scCODA^40^).

### Maternal obesity is associated with hypoxia in the placenta

**Figure 2:**
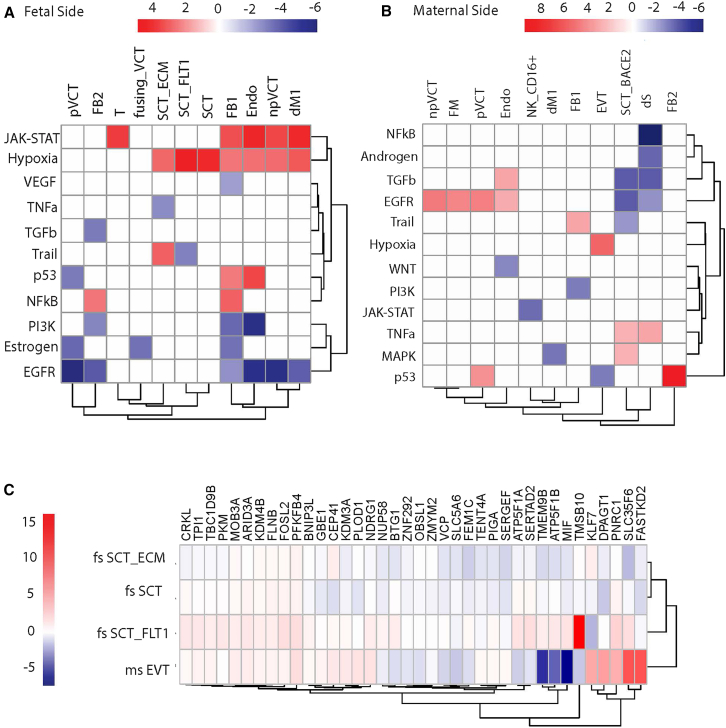
Maternal and fetal sides of placenta collected in pregnancies affected by MO are hypoxic (A and B) Pathway enrichment of genes differentially expressed by BMI on the fetal and maternal sides, based on PROGENy, showing enrichment for increased hypoxia response genes in multiple cell types. All non-zero scores shown are significant with a false discovery rate (FDR) < 0.05. (C) Genes from the hypoxia pathway in PROGENy and their differential expression by BMI, as represented by the NEBULA coefficient, in four cell types (maternal side EVTs, fetal side [fs] SCTs, fs SCT_FLT1, fs SCT_ECM) with the highest enrichment for hypoxia. Genes were selected as the top 10 hypoxia genes associated with BMI in each of the four cell types. Numerical results in Table S3. See Figure 1 legend for cell types. For all (A–C) red represents positively associated with/increased in MO, and blue represents negatively associated with/decreased in MO.

We next performed analyses to identify differentially expressed genes (DEGs) by MO status in each cell type on each side separately. We compared MO samples to lean samples, accounting for biological and technical covariates (fetal sex, maternal age, delivery mode, and sequencing batch, see STAR Methods). We relied on decoupleR^41^ to detect the enrichment of DEGs in the 14 core pathways available in PROGENy^42^ assembled from a large compendium of publicly available perturbation experiments (Figures 2A and 2B). PROGENy outperforms other pathway enrichment methods and considers transcriptional targets of signaling cascades since post-translational modifications or other molecules involved in signaling might not show transcriptional changes.^42^ Among all pathways, hypoxia was enriched (FDR<0.05) among DEGs in the most cell types on both sides. On the fetal side, hypoxia was most strongly enriched in DEGs of SCTs and SCT_FLT1 subtype (Figure 2A), and on the maternal side, hypoxia was most strongly enriched in DEGs of EVTs. MO was primarily associated with higher hypoxia in most cell types, with detected pathway enrichment, although a few cell types on the maternal side showed enrichment in the opposite direction. Of the three cell types in which MO is associated with lower hypoxia, two (Endo_M and NK_CD16+) are of maternal origin (Figure S3). Together, these results show a strong hypoxia response of the placenta in the MO condition, primarily by cells of fetal origin.

We then explored which genes were relevant for hypoxia across the different cell types. We focused on the cell types whose MO-associated DEGs had the strongest enrichment for hypoxia: maternal side (ms) EVTs, and fetal side (fs) syncytiotrophoblasts (SCTs) and two SCT subtypes on the fs: SCT_FLT1s, and SCT_ECMs (Table S2). For each cell type we identified the top 10 hypoxia associated genes, i.e., among the genes that are in PROGENy’s hypoxia pathway, the genes with the lowest *p*-value for association with maternal BMI for each cell type. This resulted in 33 genes across the four cell types, as there was some overlap. We clustered the cell types and genes by their association with MO (DESeq2 test statistic) and found that the ms EVTs were clustered separately from the rest. Although there were some genes with the consistent direction of association with MO across all cell types (Figure 2C), the top ten genes associated with hypoxia in ms EVTs were not expressed in the rest of the cell types (Table S3). These results suggest cell-type specific hypoxia responses, especially in EVTs.

### Hypoxia in the placenta is associated with short- and long-term offspring development

**Figure 3:**
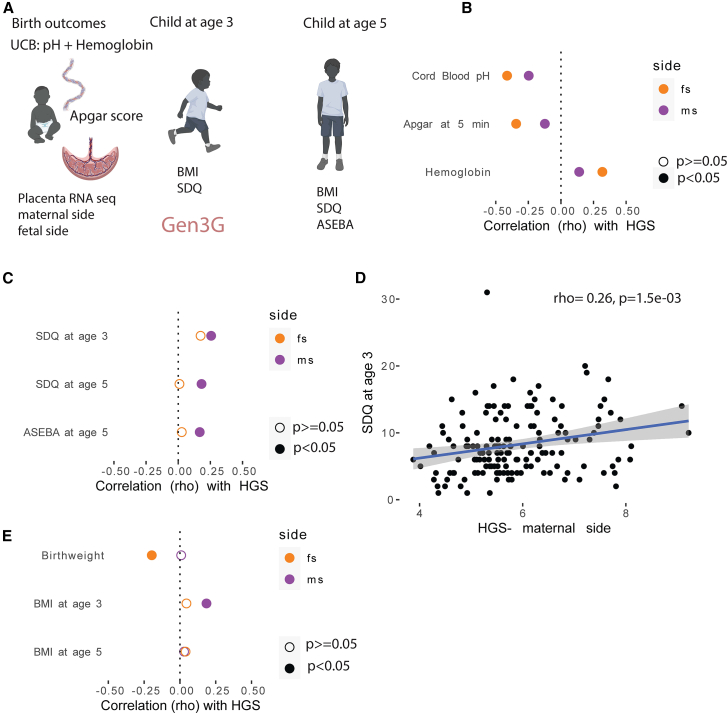
Hypoxia on the maternal (ms) and fetal sides (fs) has both shared and unique correlations to offspring outcomes (A) Overview of characteristics profiled in Gen3G relevant to this study. UCB: Umbilical cord blood. (B) Acute birth outcomes that are correlated with the placental hypoxia gene scores (HGS) derived from bulk tissue RNA-seq, with a stronger correlation on the fetal side. (C) Placental HGS calculated from biopsies on the maternal facing side is associated with three neurobehavioral outcomes: Strengths and difficulties questionnaire (SDQ) at ages 3 and 5 and Achenbach System of Empirically Based Assessment (ASEBA) at age 5. (D) Visualization of the correlation between placental HGS from biopsies on the maternal facing side and offspring SDQ score at age 3. (E) Correlations between placental HGS and growth-related traits.

Because we observed hypoxia enrichment among cell-type specific DEGs related to MO, we wanted to understand the biological relevance to the offspring of this placental hypoxia-related gene expression. We leveraged Gen3G prospective cohort with RNA-seq data from bulk placental tissue collected at delivery, in addition to birth outcomes, and child outcomes at ages 3 and 5 (Figure 3A; Table S4). We used PROGENy^42^ to calculate the hypoxia gene score (HGS) in samples from both sides of the placenta in Gen3G, based on a weighted sum of the expression of hypoxia-associated genes (see STAR Methods). We first investigated if the placental HGS was correlated with cord blood pH, as a reflection of fetal hypoxia.^43^ If hypoxia is sustained, fetal production of hemoglobin can increase as a compensatory mechanism to increase oxygen delivery,^44^ so we also investigated cord blood hemoglobin levels. We found that the HGS on both sides of the placenta was significantly correlated with cord blood PH and hemoglobin levels, and the correlations were stronger from the fetal-facing side biopsies (Figure 3B; Table S5). We also tested for a correlation between the HGS and Apgar score at 5 min post-delivery, as a measure of fetal distress and potential consequence of fetal hypoxia at the time of delivery. We again found a significant correlation with HGS on both sides of the placenta, and a stronger correlation on the fetal side (Figure 3B; Table S5). Together, the correlations between the placental HGS and birth outcomes confirmed that it is a meaningful measure of biological hypoxia.

We next selected childhood outcomes associated with higher maternal BMI in previous studies, namely impaired neurodevelopment^10^^,^^45^ and metabolic dysfunction,^1^ to examine their relationship to the placental HGS. For neurodevelopment, we relied on two independent questionnaires: SDQ (Strength and Difficulties Questionnaire)^46^ and ASEBA (Achenbach System of Empirically Based Assessment).^47^ Higher scores on both the SDQ and ASEBA suggest more abnormal behaviors.^46^^,^^47^ We found that higher placental HGS on the maternal side only was consistently correlated with higher scores of neurobehavioral testing at both ages 3 and 5, for all standardized questionnaires (Figures 3C and 3D; Table S5). The HGS derived from the fetal side of the placenta did not show a significant correlation for any of these outcomes. There was no consistent direction of association between childhood growth measures (birthweight, BMI at age 3, and BMI at age 5) of the offspring and placental HGS on either the maternal side or the fetal side (Figure 3E; Table S5), which led us to look further into neurobehavioral-related gene expression.

### Extravillous trophoblasts have the strongest signature of ND-related gene expression

**Figure 4:**
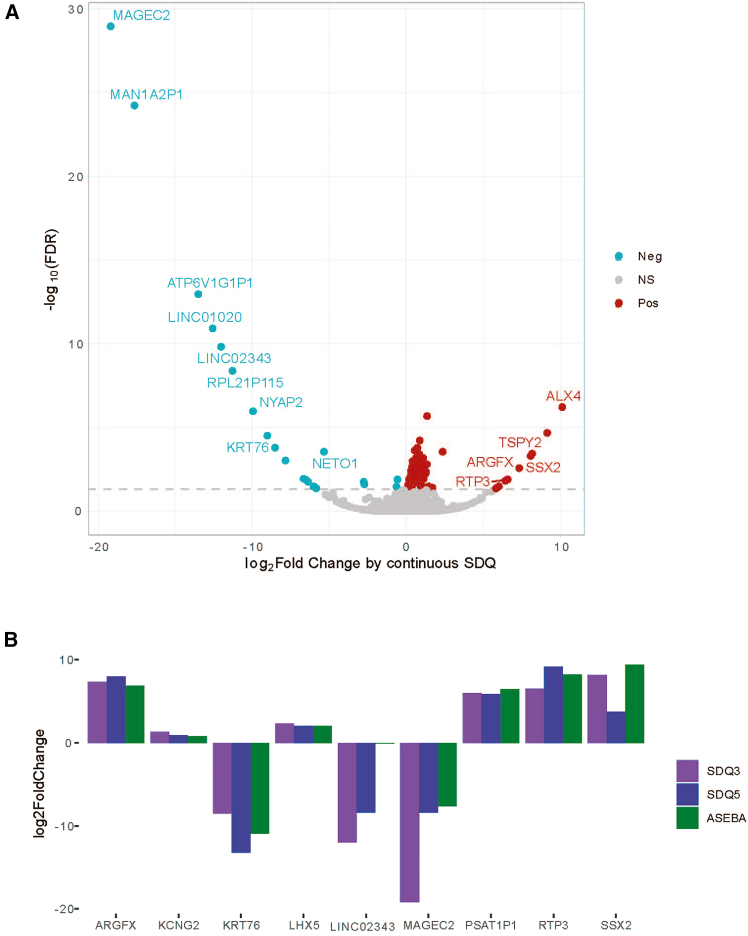
Genes whose expression on the maternal side of the placenta is associated with offspring neurobehavior (A) Volcano plot of genes associated with SDQ score at age 3. Fold change represents the fold change in gene expression associated with a unit change in SDQ 3 score. (B) All genes that were significant (FDR< 0.01) for the association with SDQ score at age 3 and validated by also being associated with SDQ score at age 5 and/or ASEBA score at age 5 (FDR< 0.01, consistent direction). FDR = False discovery rate; SDQ = Strengths and difficulties questionnaire; ASEBA = Achenbach System of Empirically Based Assessment.

To better understand the connection between hypoxia and ND, using Gen3G placental RNA-seq data, we performed a transcriptome-wide study to identify genes whose expression on the maternal side of the placenta was associated with offspring SDQ score at age 3, correcting for key potential confounders such as maternal age and maternal education (see STAR Methods, Figure 4A). We then similarly calculated genes whose expression was associated with offspring SDQ score at age 5 and ASEBA score at age 5. Several genes that were associated with the SDQ score at age 3 (FDR<0.05) were validated by their association with the SDQ score at age 5 and/or the ASEBA score at age 5 (FDR <0.05, the same direction of fold change) (Figure 4B).

**Figure 5:**
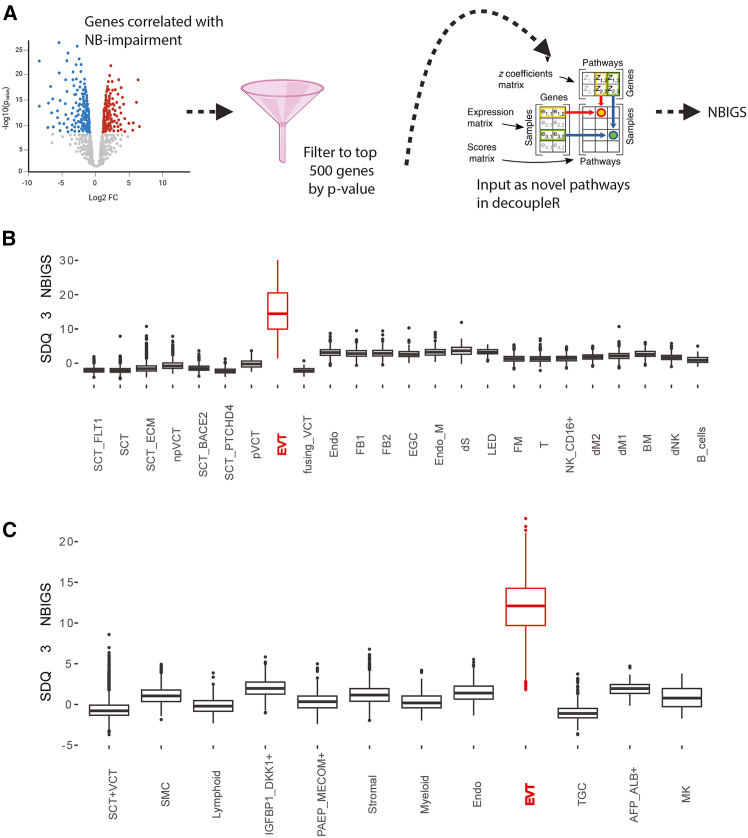
EVTs are a key cell type connecting hypoxia and neurobehavioral impairment gene scores (NBIGS) (A) Overview of the generation of NBIGS scores for SDQ at age 3, SDQ at age 5, and ASEBA at age 5. (B) SDQ 3 gene score by cell type in the single-nucleus RNA-seq dataset, showing the highest expression in EVTs. See Figure 1 legend for cell type acronyms. (C) SDQ 3 gene score by cell type in a previously published second trimester placenta single cell dataset. The original cell types annotated by the authors are used. SCT, VCT, EVT, and Endo correspond to the same cell types identified in this article. Lymphoid encompasses B cells, T cells, and NK cells. Myeloid encompasses all macrophages. MK = megakaryocytes. Please see Cao et al.^33^ for an explanation of the remaining cell types. SDQ = Strengths and difficulties questionnaire; ASEBA = Achenbach System of Empirically Based Assessment. For all boxplots, the center line is the median, the upper box is the 75^th^ percentile, and the lower box is the 25^th^ percentile.

We created placental neurobehavioral impairment gene scores (NBIGSs), using the decoupleR^41^ package. The NBIGSs are a weighted sum of gene expression, with weights based on the association between placental gene expression and each ND outcome in Gen3G, such that genes positively associated with SDQ and/or ASEBA scores in the offspring are positively weighted, and genes negatively associated with the ND scores are negatively weighted. Genes with stronger associations with ND scores had larger (absolute value) weights (see STAR Methods, Figure 5A). We calculated these placenta NBIGSs for all nuclei in the sn-RNA seq cohort and grouped by cell type to identify cell types with the highest NBIGS. EVTs stood out clearly as the cell type with the highest NBIGSs for SDQ at age 3 (Figure 5B). The results ASEBA at age 5 were highly consistent (Figure S7). Although the results for SDQ at age 5 were noisier, EVTs still had high expression compared to other cell types (Figure S9). These patterns were present in nuclei from both MO and lean conditions (Figure S10) and both fetal sexes (Figure S11).

We turned to third independent placenta dataset composed of a single-cell RNA-seq data from second trimester placenta^33^ to test the robustness of our observations that EVTs had the highest expression of NBIGSs, compared to other placenta cell types. We confirmed that EVTs had the highest NBIGSs expression (Figure 5C), and this was consistent across all three NBIGS (derived from SDQ at age 3, SDQ at age 5, and ASEBA at age 5) (Figure S12). This confirmed that EVTs have the highest expression of genes associated with offspring neurobehavior.

### Hypoxia induces the increased expression of neurobehavioral impairment gene scores in extravillous trophoblasts

**Figure 6:**
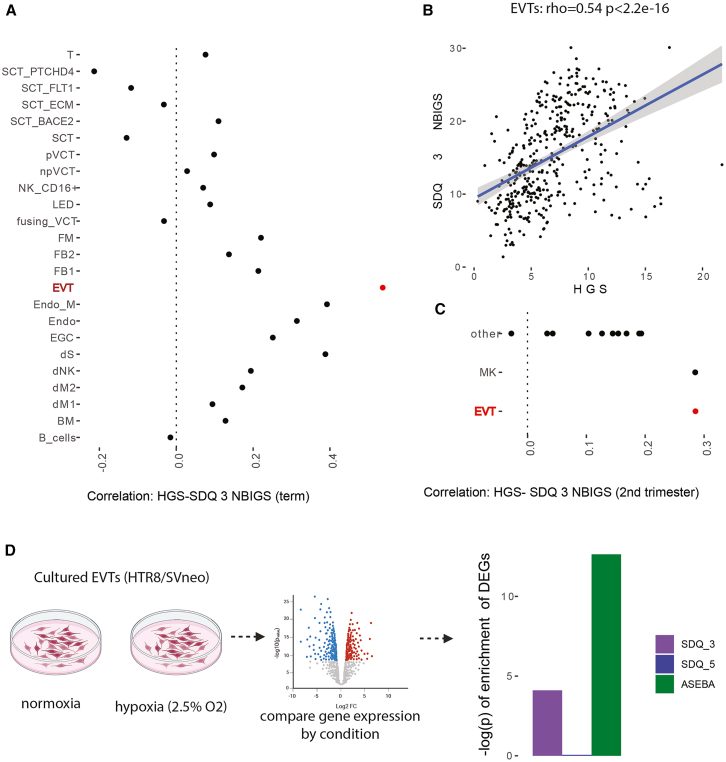
Hypoxia in EVTs increases the expression of genes associated with impaired neurobehavior (A) Correlations between SDQ 3 NBIGS and HGS across cell types in our term single nucleus RNA-seq. (B) SDQ 3 gene score and hypoxia gene score are correlated in EVTs (highlighted in red). (C) Correlations between SDQ 3 gene score and HGS across cell types in previously published second trimester single cell RNA seq. MK = Megakarocytes. (D) Exposing cultured HTR-8/SVneo cells to hypoxia induces gene expression enriched for NBIGSs, reanalyzing previously published data.^48^

We tested if placenta NBIGSs were correlated with placenta HGS in each cell type in both our term sn-RNA seq and the published sc-RNA seq from second trimester placenta.^33^ EVTs stood out as having the highest correlation in our term single-nucleus dataset across all three NBIGSs (Figure 6A; Table S6). The correlation between HGS and NBIGS in EVTs was highly significant (Figure 6B; SDQ 3 rho = 0.54 *p* < 2.2e-16; Table S6). This was also true for the second trimester dataset (Figure 6C; Table S7). Together, the results from two independent sc/sn-RNA seq datasets at different time points in gestation are consistent with the correlation found in Gen3G between placental HGS and phenotypic ND outcomes, and goes further to identify EVTs as a key cell type in this correlation.

Given our findings in EVTs, we leveraged a dataset of HTR-8/SVneo cell line derived from invasive trophoblasts, an *in vitro* model of EVTs. Previous work exposed cultured trophoblasts to either hypoxia or normoxia conditions and performed bulk RNA-seq to calculate DEGs comparing the two conditions.^48^ We checked for enrichment among these hypoxia-DEGs and found a significant enrichment of genes included in NBIGSs (SDQ-3 enrichment: *p* = 7.9e- 5, ASEBA-5 enrichment: *p* = 2.3e-13, Figure 6D), with higher expression in the hypoxia condition (positive test score statistics of 3.95 for SDQ-3 and 7.34 for ASEBA-5). This shows that exposing human EVTs to hypoxia increases the expression of genes associated with impaired neurobehavior, and supports observations from the analyses we performed in Gen3G and sn/scRNA-seq placental datasets.

### Hypoxia attenuates the relationship between maternal body mass index and ND score in extravillous trophoblasts

**Figure 7:**
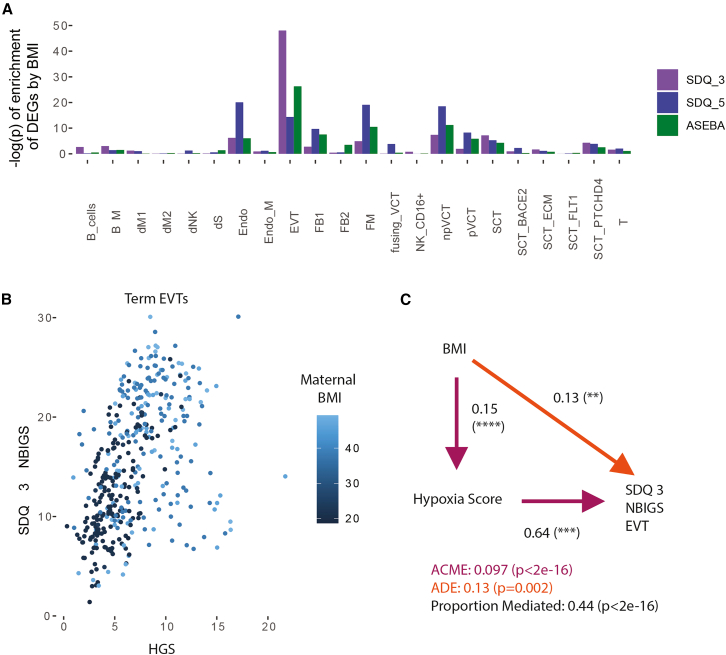
DEGs by BMI are enriched for genes associated with neurobehavior, and hypoxia potentially links BMI to neurobehavior (A) Enrichment of neurobehavioral-impairment associated genes among each cell type DEGs by BMI, on the maternal side. (B) Three-way relationship between BMI, hypoxia, and SDQ 3 NBIGS in EVTs. (C) Accounting for hypoxia response gene expression attenuates the relationship between BMI and NBIGSs in EVTs, for SDQ at age 3. ACME: Average causal mediated effect ADE: Average direct effect. NBIGS: Neurobehavioral Impairment Gene Score.

After exploring associations between placental gene expression scores of hypoxia and ND in EVTs, we return to MO, which initially highlighted hypoxia. Since EVTs were the cell type in which MO-related DEGs were the most enriched for hypoxia on the maternal side, it is possible that maternal BMI, placental hypoxia, and offspring ND are all connected. We first checked for the enrichment of the NBIGSs among MO-related DEGs in the term sn-RNA data. EVTs stood out as the cell type whose MO-related DEGs had the most strongly significant enrichment for NBIGSs (Figure 7A). DEGs in fetal macrophages and endothelial cells also showed enrichment, albeit to a lesser extent. This shows that genes associated with MO are significantly enriched for genes associated with ND, and that this enrichment is strongest in EVTs.

We then examined the associations between continuous maternal BMI placental HGS, and NBIGS in the term sn-RNA EVTs. We found that continuous maternal BMI was correlated with both the HGS (rho = 0.51, *p* < 2.2e-16) and SDQ 3 NBIGS (rho = 0.37, *p* < 2.2e-16). As described in Figure 5C, the HGS and NBIGS correlated in term EVTs (rho = 0.54, *p* < 2.2e-16). To visualize these three pairwise correlations together, we plotted the HGS with SDQ 3 NBIGS, colored by maternal BMI (Figure 7B). We then performed mediation analysis^49^ to estimate the indirect effect of BMI on NBIGS through HGS, as well as the direct effect between BMI and NBIGS that remains when accounting for HGS. The indirect effect was 0.097. Using bootstrapping, we found the indirect effect to be significant (95% confidence interval: 0.0632–0.13; *p* < 2.2e-16; Figure 7C). 44% of the total association between BMI and NBIGS (SDQ at age 3) in EVTs was attributable to the indirect effect. The associations with SDQ and ASEAB at age 5 confirmed both a significant direct and indirect association of BMI and NBIGSs, although the proportion of the indirect effect was lower (10% and 25% respectively). Together, this shows that a substantial proportion of the effect of BMI on neurobehaviorally relevant genes in EVTs may be mediated by placental (mostly EVT) hypoxia.

## Discussion

In the current study, we showed that gene expression signature related to hypoxia in EVTs was associated with both maternal obesity and child neurobehavior in humans. We rely on three independent cohorts with placental RNA seq data to test our hypotheses and more robustly support our conclusions, including two single-cell and single-nucleus RNA seq datasets from different time points in gestation, and one large cohort with bulk RNA seq from term placenta with offspring neurobehavioral measures (Gen3G). The three cohorts are geographically and demographically diverse: Gen3G is a cohort of primarily White women in Sherbrooke, Quebec^30^; the single-nucleus RNA-seq generated in this study is from placenta of mostly Black women in St. Louis, Missouri, and the previously published second trimester dataset comes from the University of Washington.^33^ The consistency of findings across these three cohorts increases the generalizability of our study.

Maternal obesity has been associated with fetal hypoxia as indicated by higher cord blood erythropoietin concentrations^50^ and hematocrit,^44^ which are thought to be compensatory mechanisms. In mice, offspring of high-fat diet (HFD) dams have markers of oxidative stress in the placenta^51^ and, separately, in the fetal brain,^52^^,^^53^ but the long-term consequences on the offspring of hypoxia associated with MO/HFD have not been studied in mice. Placental hypoxia, in the context of maternal uterine artery ligation in rodents, has been linked with structural changes in the offspring brain^54^^,^^55^: reduced hippocampal size,^56^ abnormal neural migration,^57^ and delayed myelination.^55^^,^^58^ We extend previous work by studying maternal obesity, placental hypoxia, and offspring neurobehavior together, and by studying transcriptome-wide gene expression in term human placenta.

To add evidence of causal mechanism by which hypoxia connects MO to offspring neurobehavior, future studies should test if the use of antioxidants can partially rescue changes in the placenta associated with MO, in animal models. Previous work in mice has shown that MO is associated with both behavioral deficits in the offspring^59^ and placental hypoxia.^51^ An antioxidant (MitoQ) has been shown to rescue placental hypoxia in rats^60^ and there is also preliminary *in vivo* evidence that mitochondrial antioxidants can ameliorate the consequences of exposure to gestational hypoxia relating to offspring neurodevelopment^60^ and cardiovascular development^61^^,^^62^ in rodents. Future experiments could test if the placental injection of antioxidants rescues behavioral outcomes of offspring from HFD dams, which would causally implicate placental hypoxia.

Recent advances in single-cell technology have allowed for easier profiling of numerous cell types simultaneously. We generate one of the largest transcriptomic databases of human SCTs, and the only dataset to explore transcriptomic changes associated with MO in trophoblasts more generally. Our unbiased profiling of all cell types present demonstrated that EVTs consistently stood out as the cell type with the highest expression of NBIGSs and with the strongest correlation between HGS and NBIGSs. Given that EVTs are primarily on the maternal side of the placenta, this could explain the null relationship seen on the fetal side between HGS and neurobehavioral outcomes in Gen3G. A small subset of EVTs appeared to be resilient to hypoxia, and future studies focused on EVTs through methods such as flow cytometry sorting based on EVT-markers could further investigate this population.

SLC2A3, one of the top genes dysregulated in cultured EVTs in response to hypoxia, is thought to be the glucose channel primarily responsible for *trans*-placental (maternal to fetal) glucose transport (as opposed to intra-placental glucose transport).^63^ Copy number variants of SLC2A3 has been associated with attention deficit hyperactivity disorder and bipolar disorder.^64^ Improper glucose transport could have large effects on brain development. Two other genes were notable: CEBPB was one of eight key transcription factors enriched for genes perturbed in blood and brain tissue of people with ASD^65^ and HMOX1 has been associated with neuroinflammation in rats.^66^ Tissue specific knock-out animal models could further investigate the effects of candidate genes in the placenta and the brain separately.

EVTs’ primary known function is to remodel the maternal spiral arteries in early pregnancy,^67^ and their role in mid-to-late pregnancy is largely unknown. EVTs are highly sensitive to oxygen levels—hypoxia promotes EVT differentiation from stem cell precursors through HIF-dependent modulation.^67^ It is possible that hypoxia, a normal condition in early pregnancy, aberrantly activates EVTs in the second and third trimesters, leading to changes relevant for neurobehavior of the offspring. Synaptogenesis begins around week 16 of gestation and continues past birth,^45^ and myelination occurs starting from a few weeks before term delivery.^45^ These key processes of neurodevelopment take place in the second half of pregnancy, and might be affected by oxidative stress. Transcriptome wide association studies (TWAS) identified 139 placenta schizophrenia-specific risk genes, and of those TWAS hits predicted to be upregulated in schizophrenia, there was significant enrichment for expression in EVTs.^14^ Emerging evidence from Mendelian randomization supports a causal role of trophoblast physiology (and inflammation from fetal macrophages) in the development of offspring depression.^27^^,^^28^ In particular, EVTs and villous cytotrophoblasts were significantly enriched for genes whose variants are associated with offspring depression, and hypoxia is a postulated mechanism.^28^ Cultured trophoblast cells exposed to hypoxia release factors into media that caused damage to neurons, including decreased dendrite length.^60^ In particular, hypoxia affects extracellular vesicles (EVs) derived from EVTs.^68^^,^^69^ There is growing interest in how affect the developing brain and blood brain barrier integrity affected by EVs.^70^ One could isolate EVs non-invasively from cord blood, and sort based on HLA-G, a marker of EVTs.^31^ The contents of these EVs could be examined for neurotransmitter content, microRNA species, and other soluble factors that might explain their connection to the brain.

### Limitations of the study

Although the sc/sn-RNA-seq cohorts have tens of thousands of cells/nuclei, they come from a small number of unique mother-child pairs. This limitation is in part compensated by the corroboration of findings in the large bulk cohort, Gen3G. Our sample size precluded us from examining sex-specific differences in mechanisms, and future studies with larger sample sizes could investigate this. As a primarily observational study with human tissue, we are unable to prove causality, beyond the increase in neurobehavioral gene expression following cultured trophoblast’s exposure to hypoxia.

## Resource availability

### Lead contact

Requests for further information and resources should be directed to and will be fulfilled by the lead contact, Eunjung Alice Lee (ealee@childrens.harvard.edu).

### Materials availability

This study did not generate new unique reagents.

### Data and code availability

• Data: Single-nucleus RNA-seq data generated in this study is publicly available at GEO: GSE271976. Single-cell RNA-seq data from Cao et al.^33^ is publicly available at GEO: GSE156793. Gen3G is protected data available via application on dbGaP: phs003151.v1.p1.
• Code: All original code has been deposited on GitHub: https://github.com/99fatimagr/MO-placenta and is also publicly available on Zenodo: https://doi.org/10.5281/zenodo.14647966.
• Additional information: Any additional information required to reanalyze the data reported in this article is available from the lead contact upon request.

## Acknowledgments

We thank the Women and Infants Health Specimen Consortium (WIHSC) Biobank at Washington University School of Medicine for the placental samples used to generate new data for this study, and thank all participants who provided placental samples referenced in this study. We thank Diane Shao for her guidance on frozen tissue handling and snRNA-seq data generation. This work was supported by the 10.13039/100000002National Institutes of Health (NIH) (DP2 AG072437), the Suh Kyungbae Foundation, and the 10.13039/100017033Allen Discovery Center program, a Paul G. Allen Frontiers Group advised program of the Paul G. Allen Family Foundation. RNA sequencing in Gen3G was supported by a grant from the 10.13039/100000002NIH (R01HD094150). Gen3G was initially supported by a 10.13039/501100000156Fonds de Recherche du Québec - Santé (FRQS) operating grant (grant #20697); 10.13039/501100000024Canadian Institutes of Health Research (CIHR) operating grants (grant #MOP 115071 and #PJT-152989). P.-E.J. in a senior research scholar from the Fonds de recherche du Québec—Santé. The RNA-seq analyses from Gen3G data was enabled in part by support provided by Calcul Québec and the 10.13039/501100021202Digital Research Alliance of Canada (alliancecan.ca). Biorender was used to create Figures 1A, 3A, 5A, and 6D.

## Author contributions

F.G.-R. and E.A.L. designed the study, with clinical guidance from R.M.R. and M.-F.H. F.G.-R. generated the single-nucleus RNA-seq data. F.G.-R. performed the analyses, with help from S.M. and F.W. F.G.-R. and S.M. had unrestricted access to all data. M.-F.H. and P.-E.J. supervised analyses with Gen3G data. R.M.R. and E.A.L. supervised single-nucleus RNA-seq analyses. F.G.-R. wrote the article, with substantial contributions from M.-F.H. and R.M.R. All authors agreed to submit the article, read and approved the final draft and take full responsibility of its content, including the accuracy of the data.

## Declaration of interests

The authors declare no competing interests.

## STAR★Methods

### Key resources table

**Table:** 

REAGENT or RESOURCE	SOURCE	IDENTIFIER
**Biological samples**		

Placenta samples	Women and Infant Health	
	Specimens	
	Consortium, Washington University	

**Deposited data**		

Single nucleus RNA-seq of maternal and fetal sides of term placenta	This study	GEO: GSE271976
Single nucleus RNA-seq of second trimester placenta	Cao et al.^33^	GEO: GSE156793
Gen3G cohort	Hivert et al.^71^	dbGap: phs003151.v1.p1

**Software and algorithms**		

PROGENy	Schubert et al.^42^	https://github.com/saezlab/footprints
NEBULA h-likelihood (HL) algorithm	He et al.^72^	https://github.com/lhe17/nebula
Original code for analysis and figure generation	This study	Zenodo: https://doi.org/10.5281/zenodo.14647966
Seurat 4.4.0	Hao et al.^73^	https://github.com/satijalab/seurat
CellRanger 6.0.1/6.1.2	10X Genomics	https://support.10xgenomics.com/single-cell-gene-expression/software/pipelines/latest/installation
CellBender 0.3.0	Fleming et al.^74^	https://github.com/broadinstitute/CellBender
scDblFinder 1.15.4	Germain et al.^75^	https://github.com/plger/scDblFinder
singleR 2.2.0	Aran et al.^76^	https://github.com/dviraran/SingleR
gProfiler	Raudvere et al.^77^	https://biit.cs.ut.ee/gprofiler
scCODA	Büttner et al.^40^	https://github.com/theislab/scCODA
DESeq2 1.40.2	Love et al.^78^	https://www.bioconductor.org/packages/release/bioc/html/DESeq2.html
decoupleR 2.6.0	Badia-i-Mompel et al.^41^	https://www.bioconductor.org/packages/release/bioc/html/decoupleR.html
pheatmap	RPackage	https://cran.r-project.org/web/packages/pheatmap/index.html
mediation 4.5.0	Tingley et al.^49^	http://cran.r-project.org/package=mediation

### Experimental model and study participant details

No new participants were recruited for this study. The samples acquired from Washington University were approved under the IRB 201013004, and demographic information on the samples included in the analysis is available in Table S1. This includes 10 maternal-facing samples and 10 fetal-facing samples. The samples analyzed from the Gen3G cohort were approved under the Harvard Pilgrim Health Care IRB 1288881-13, and the demographic information for these samples is accessible through dbGap: phs003151.v1.p1. This includes 262 maternal-facing samples and 83 fetal-facing samples. Samples were categorized into groups using 18.5–25 BMI range for the lean group and >35 BMI for the MO group.

### Method details

#### Generation of single-nucleus RNA seq data

Human placenta samples were obtained at Washington University in St. Louis, as a part of the Women and Infant Health Specimens Consortium, following institutional review board approval (IRB 201013004). For this study, we selected term, singleton pregnancies that were not complicated by any maternal autoimmune disorder, active infection, substance abuse, preeclampsia, gestational hypertension, or placental abruption. Additionally, no mothers had pre-existing or gestational diabetes, and all mothers were less than 35 years old. All samples in the lean category had a pre-pregnancy BMI within 18.5–25 and all MO samples had a BMI greater than 35. Samples were stored frozen at −80°C until use. The maternal side was sampled by slicing a thin 10mm layer off maternal facing side of the placenta (including both villous tissue and basal plate), following peeling back of the chorioamniotic membranes. The fetal side was sampled by taking the spongy tissue located directly under the basal plate, making sure to exclude any basal plate.

Nuclei were isolated from placental samples following a modified version of the Allen Brain institute protocol for nuclei extraction. Briefly, tissue was homogenized using an electric pulsator and via dounces in a glass tissue homogenizer. A series of two washes was used to remove debris, and then nuclei were stained with DAPI. Flow cytometry was used to count 10,000 nuclei per sample, with gating based on DAPI-positive cells. The standard Chromium 10X v3.1 kit instructions were followed to generate the single-nucleus RNA-seq data. CellRanger was used to align sequencing reads to hg38 (CellRanger 6.0.1 for the four pilot samples in batch 1, and 6.1.2 for the rest of the samples. All batches had representation from both MO and lean groups). CellBender 0.3.0^74^ was used to remove ambient noise from droplets with nuclei, and to identify empty droplets. Seurat 4.4.0^73^ was used to perform filtering (gene count >200, UMI count> 500, mitochondrial reads percentage <5), integrate samples accounting for batch, and perform clustering. scDblFinder 1.15.4^75^ was used to identify doublets, and all nuclei called as doublets were removed, as well as clusters that were dominated by doublets. Initial clustering identified 3 major cell types (trophoblasts, immune cells, and endothelial-stromal cells). Within each large cluster, nuclei were re-integrated and re-clustered to improve performance and identification of rarer cell types. singleR 2.2.0^76^ was used to map to existing clusters from Vento-Tormo et al.,^31^ and Seurat FindMarkers was used to identify markers of novel SCT subclusters. Markers were compared with Wang et al.^22^ gProfiler^77^ was used to calculate pathway enrichment of SCT sub-clusters. scCODA was used to test differential cell type abundance by BMI condition, adjusting for the covariates fetal sex, delivery mode, sequencing batch, and maternal age, for maternal side samples and fetal side samples separately.

#### Pathway enrichment among DEGs in sn-RNA seq

The NEBULA^72^ h-likelihood (HL) algorithm was used to calculate differentially expressed genes (DEGs) by maternal BMI, including maternal age, fetal sex, delivery mode, and batch as covariates for each cell type on each placental face. Pseudobulk counts were calculated for each cell type on each side of the placenta by summing counts for all relevant nuclei of an individual. For each cell type and placental side, genes with at least 10 counts across all samples were kept, and genes with less than 10 counts were removed for low expression. DEGs based on pseudobulk counts were calculated using DESeq2 1.40.2,^78^ using the same covariates. decoupleR 2.6.0^41^ was used to calculate enrichment of the 14 canonical pathways from PROGENy.^42^ We followed the guideline’s in the tutorial (https://saezlab.github.io/decoupleR/articles/pw_bk.html). We relied on the multivariate linear model (mlm) tool on the DESeq test statistic, and used the top 500 genes for each pathway in PROGENy. False discovery rate^79^ (FDR) corrections were applied to correct for multiple hypothesis testing. pheatmap^80^ was used to make all plots.

To calculate enrichment of neurobehavioral related genes among BMI DEGs, we calculated novel gene weights (see calculating genes whose expression in placenta is associated with neurobehavioral impairment), just like those that exist in PROGENy^42^ for pathways such as hypoxia. The univariate linear modeling (ulm) tool from decoupleR was used to test for enrichment of these neurobehavioral impairment genes scores (NBIGSs) among the BMI DEGs, relying on the test statistic from DESeq2.

#### Gen3G cohort

Gen3G is a prospective cohort of pregnant women at the Center Hospitalier Universitaire de Sherbrooke (CHUS), Quebec (Canada). Participants entered the study between 1 January 2010 to 30 June 2013. Non-singleton pregnancies and women with regular use of medications that influence glucose regulation were excluded from the cohort. All study participants provided informed written consent, and the study protocols were reviewed by the ethical committees from CHUS, and from the Harvard Pilgrim Health Care Institute (IRB 1288881-13). To most closely match the single-nucleus RNA seq cohort, in this study, we excluded Gen3G mother-child pairs with maternal smoking during pregnancy, preeclampsia or gestational diabetes, birth before gestational age of 37 weeks, babies born small for gestational age, or placentas that were manually evacuated. Our final sample size for the current analyses was 262 placental samples that were collected at delivery from the maternal-facing side, and 83 from the fetal-facing side (also collected at delivery).

Birthweight was measured using standard clinical procedures at delivery. Cord blood and one cm^3^ of placental tissue from maternal and fetal facing sides were collected at delivery. For more information on the Gen3G cohort and biospecimens collected at birth, please see Guillemette et al.^30^ Total RNA was extracted from placenta, collected at the time of delivery. Samples with an RNA integrity of at least 4 were sequenced (*n* = 466). For more details on pre-processing of Gen3G RNA-seq data, please see the “RNA extraction, sequencing and QC” section of Hivert et al.^71^

Trained research staff measured BMI in the children at ages 3 and 5 using standardized protocols. Mothers completed the Strengths and Difficulties Questionnaire (SDQ)^46^ for their children at ages 3 and 5, along with the Child Behavior Checklist from the Achenbach System of Empirically Based Assessment (ASEBA)^47^ at age 5. For more details on the neurobehavioral measures in Gen3G, please see Faleschini et al.^81^

#### Hypoxia gene score correlations in Gen3G

The hypoxia gene score (HGS) was calculated by applying PROGENy to the expression matrix of all placental samples in Gen3G that met exclusion criteria described above. mlm from decoupleR was used to calculate an enrichment score of each pathway in PROGENy (including hypoxia). The Shapiro-Wilk normality test (shapiro.test, base R 4.3) was used to test for normality of hypoxia scores among placental samples on the maternal side and fetal side.

Hypoxia scores on both sides were not normally distributed per the Shapiro-Wilk normality test^82^ (fetal side *p* = 6.8e-04, maternal side *p* = 5.2e-07), so we used Spearman correlations to test for associations between HGS and cord blood pH, fetal hemoglobin, Apgar score at 5 min, birthweight, neurobehavioral scores at ages 3 and 5, and offspring BMI at ages 3 and 5.

#### Calculating genes whose expression in placenta is associated with neurobehavioral impairment

We used Gen3G to calculate genes whose expression on the maternal side of the placenta was associated with offspring neurobehavioral scores (SDQ score at ages 3 and 5, ASEBA score at age 5). We used normalized (using base R scale) continuous neurobehavioral scores as the predictor, gene expression as the outcome, and included the following covariates: maternal age (normalized with base R scale), maternal education (categorical), delivery mode, fetal sex, and sequencing batch. We used consistent exclusion criteria as detailed above (“Hypoxia gene score correlations in Gen3G″). DESeq2 was used to calculate the association strength and significance. We tested associations using scores from each ND questionnaires completed at 3years and/or 5years (SDQ 3, SDQ 5, and ASEBA 5).

#### NDD score generation and application to sn/sc RNA seq

We used decoupleR (ulm) to calculate neurobehavioral impairment gene scores (NBIGSs) using the weights derived from the association described above, using the top 500 genes by *p*-value of association for each outcome. The test statistic from DESeq2 was used as the weights in decoupleR. We then applied these NBIGSs to the single nucleus (generated in this paper) and single cell (Cao et al.^33^) normalized expression matrices. This resulted in a gene score for each neurobehavioral measure (SDQ 3, SDQ 5, and ASEBA 5) for each cell/nucleus. We then grouped expression by cell type on the maternal side of the placenta in the single nucleus dataset, and all together in the Cao et al. dataset because side of origin was not available.

Next, we calculated a hypoxia gene score (HGS) similarly using decoupleR, with PROGENy as the gene sets. We performed Spearman correlations between the HGS and each NBIGS in each cell type.

We used the mediation 4.5.0^49^ package to calculate the average casual mediated effect (ACME) and average direct effect (ADE). The ACME is the estimated the indirect effect of BMI on NBIGS through HGS, and the ADE is the association between BMI and NBIGS that remains when accounting for HGS. Continuous maternal pre-pregnancy BMI was the treatment variable and HGS was the mediator.

### Quantification and statistical analysis

All statistical analysis was conducted with R version 4.3. Figures and figure legends include relevant information on statistical tests performed and corresponding values. Shapiro Wilk normality test was used to determine normality of distribution of data, and Spearman correlations were used to test the association of variables. Test statistics from DESeq, NEBULA, and decoupleR were used, and false discovery rate (FDR) correction was used when applicable. Further information on the algorithms and statistical tests performed can be found in the corresponding “method details” section and the test statistics of the utilized software are available in supplementary tables.
