# Supplementary material for: Hypoxia in extravillous trophoblasts links maternal obesity and offspring neurobehavior
Source: iScience. 2025 May 12;28(6):112636. doi: 10.1016/j.isci.2025.112636 (PMC12152354; doi:10.1016/j.isci.2025.112636)
Supplement: Document S1. Figures S1–S12 and Tables S1–S7 [file mmc1.pdf]

iScience, Volume *28*

Supplemental information

Hypoxia in extravillous trophoblasts links maternal obesity and offspring neurobehavior

Fatima Gunter-Rahman, Shayna Mallett, Frédérique White, Pierre-Étienne Jacques, Ravikiran M. Raju, Marie-France Hivert, and Eunjung Alice Lee

Figure S1: Proportions of each batch for each cell type

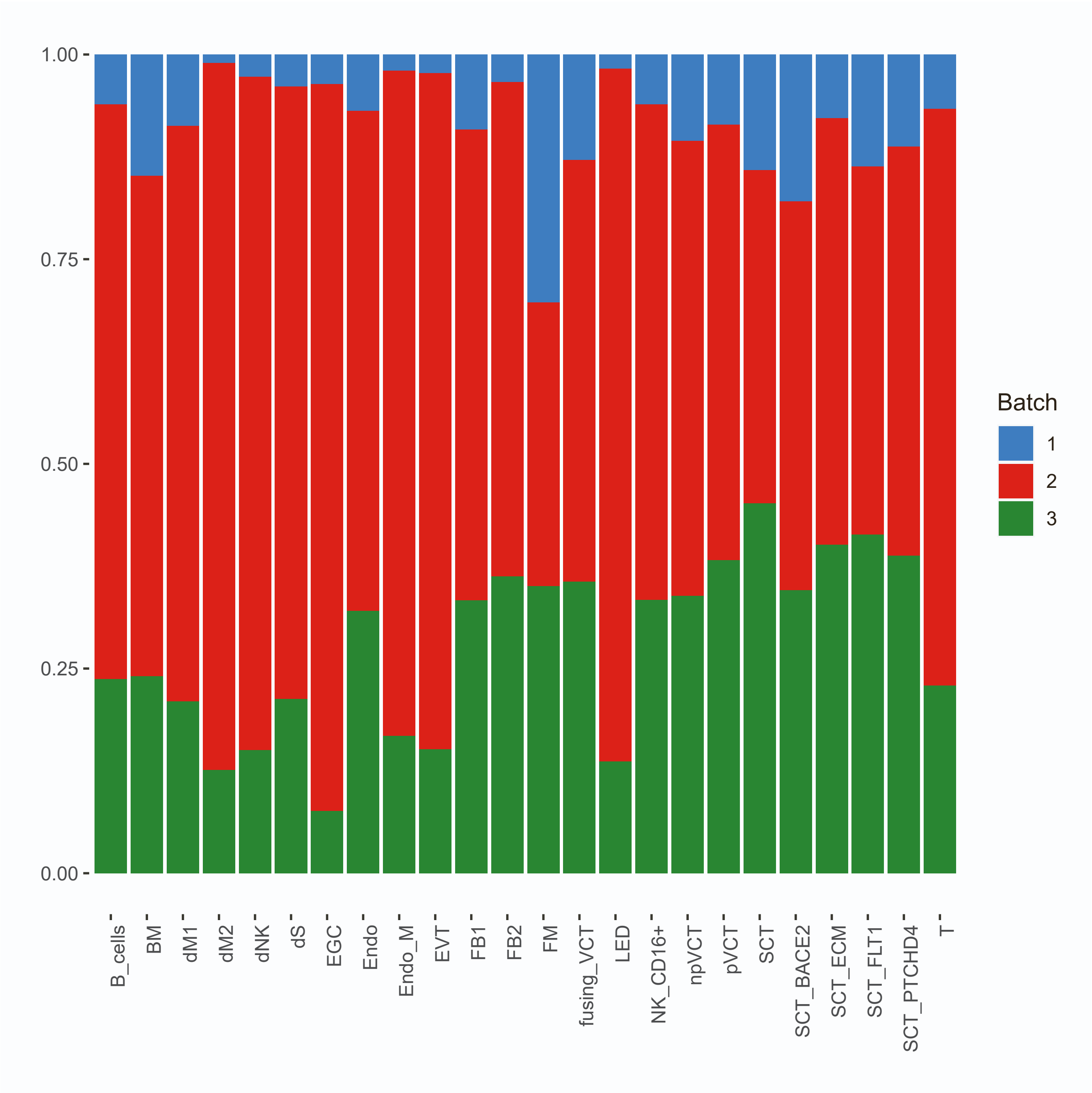

See main Figure 1 legend for abbreviations.

Figure S2: Proportions of each side (maternal facing or fetal facing) for each cell type. Side of origin is separate from person of origin (i.e. fetal or maternal DNA, as shown by cell type in Figure S3). Both sides have cell types of maternal and fetal origin.

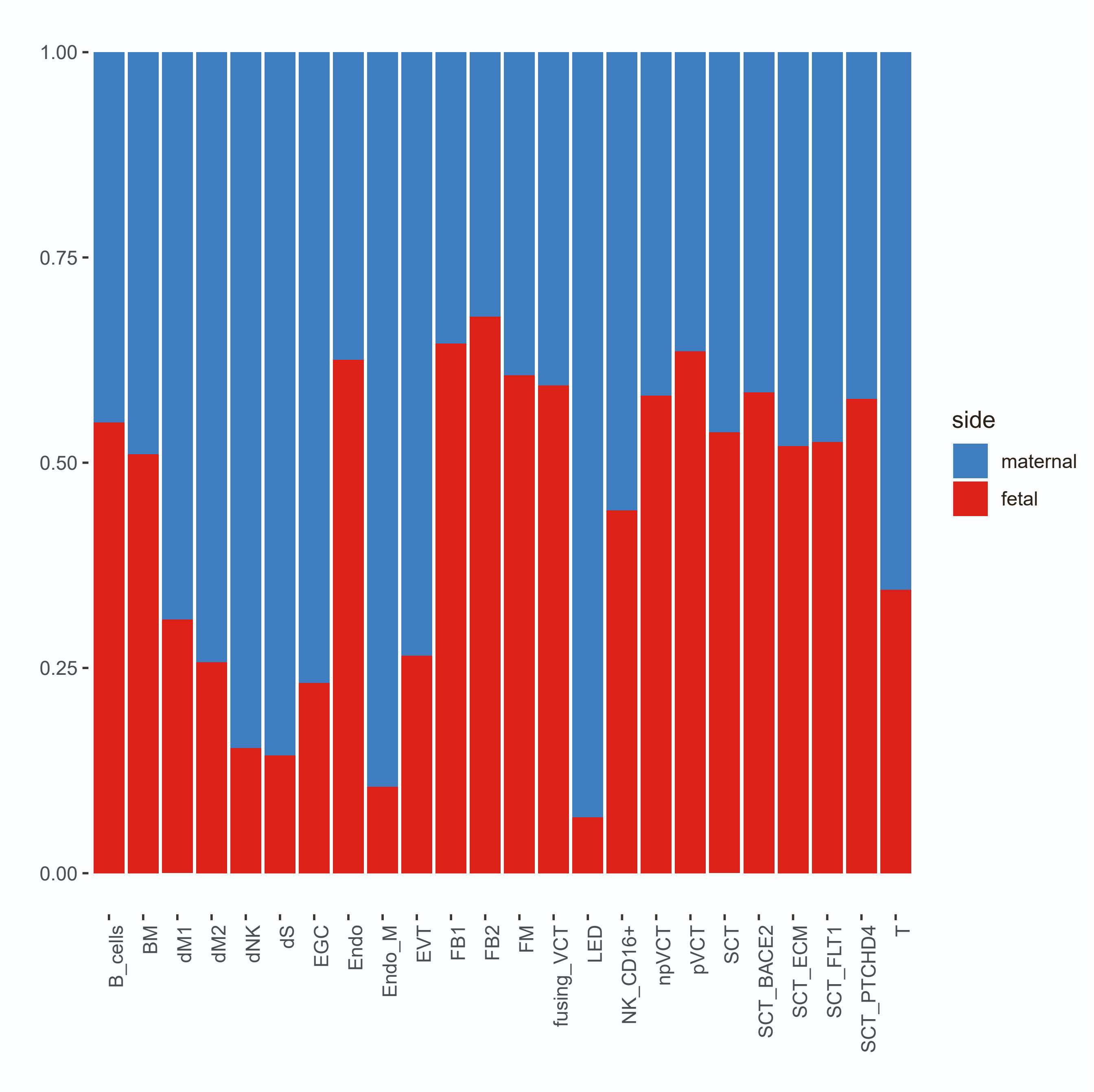

See main Figure 1 legend for abbreviations.

Figure S3: Inferred maternal/ fetal origin on cells based on sex-chromosome gene expression among male fetus placenta

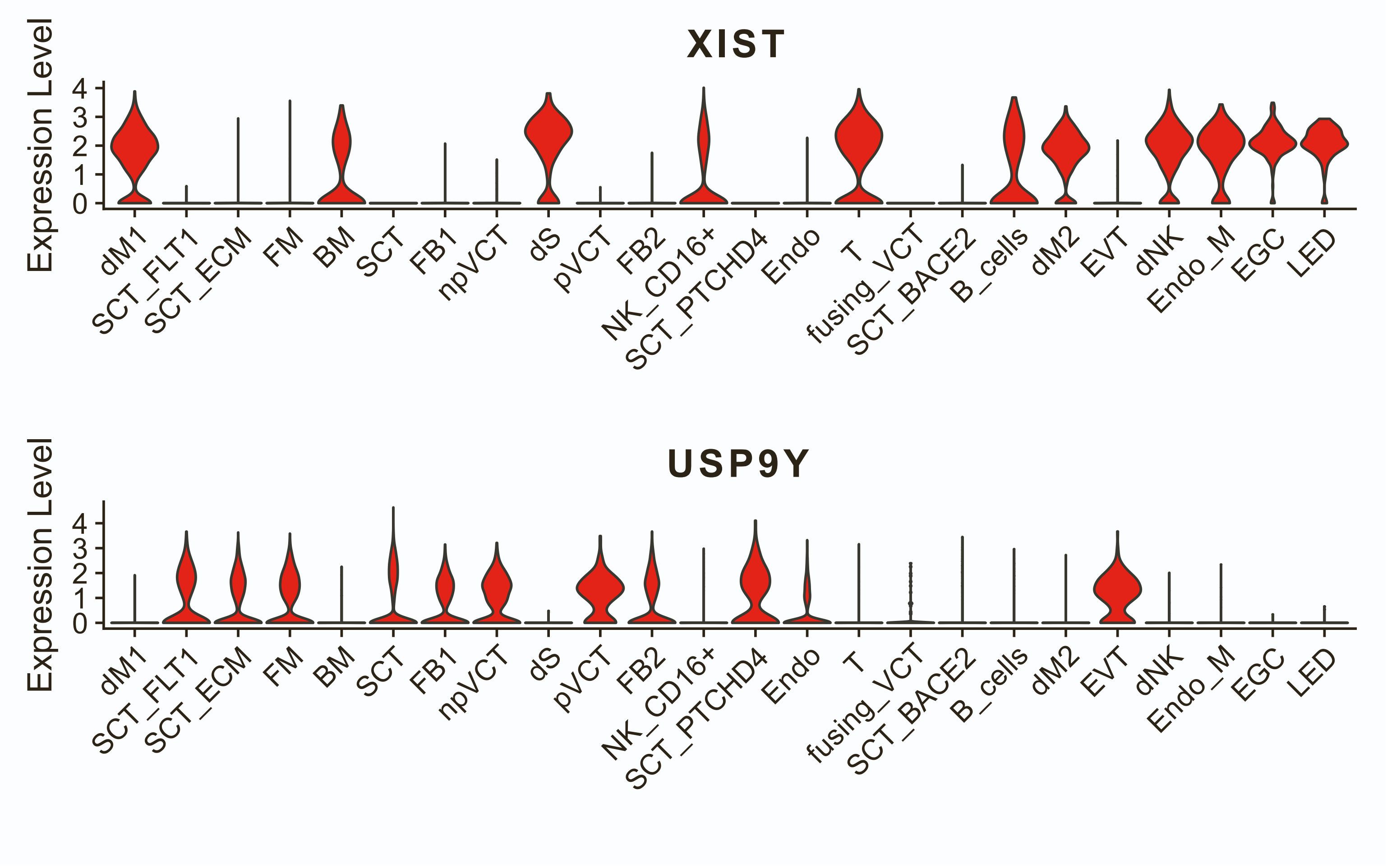

See main Figure 1 legend for abbreviations.

Figure S4. Macrophage subtype markers from previously published work.

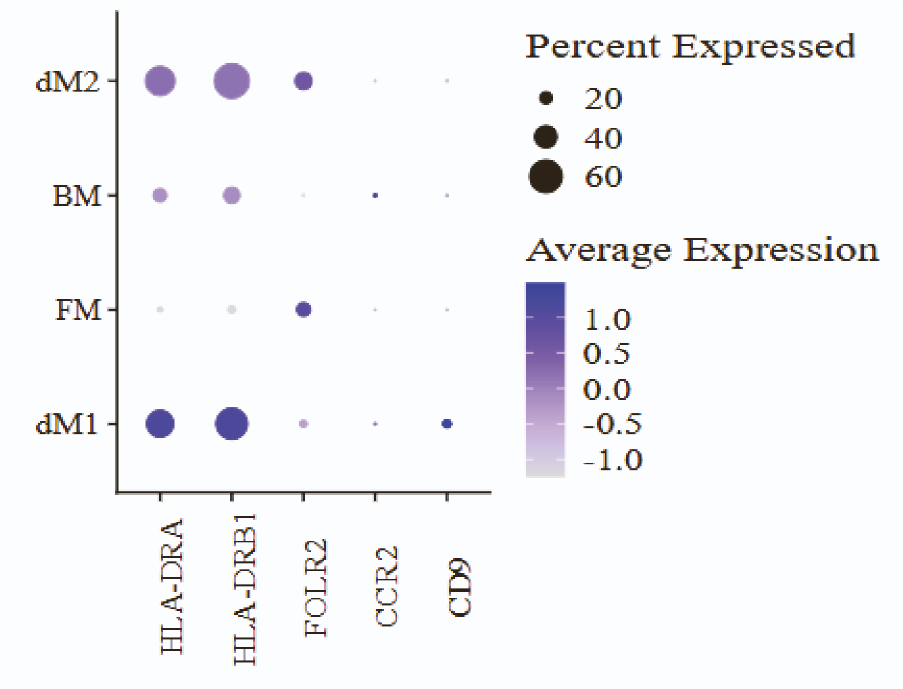

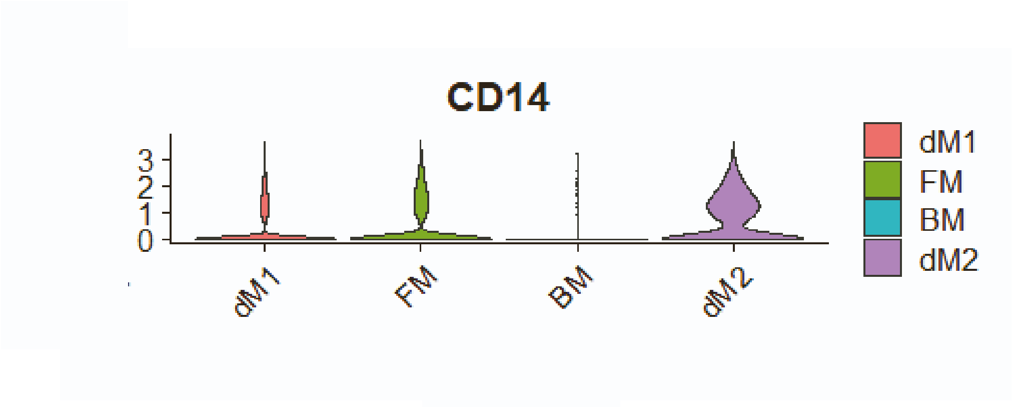

Figure S5: Gene ontology enrichment for markers of the SCT_ECM cell type. Driver terms from gProfiler are highlighted in blue.

GO:MF			stats
	Term name	Term ID	p_{adj}
☑	oxidoreduction-driven active transmembrane transpor...	GO:0015453	1.784×10^{-10}
☐	electron transfer activity	GO:0009055	3.031×10^{-8}
☑	extracellular matrix structural constituent	GO:0005201	3.433×10^{-8}
☐	primary active transmembrane transporter activity	GO:0015399	8.115×10^{-7}
☐	oxidoreductase activity	GO:0016491	1.487×10^{-5}
☐	NADH dehydrogenase (ubiquinone) activity	GO:0008137	7.715×10^{-5}
☐	NADH dehydrogenase (quinone) activity	GO:0050136	8.738×10^{-5}
☐	NADH dehydrogenase activity	GO:0003954	1.110×10^{-4}
☐	NAD(P)H dehydrogenase (quinone) activity	GO:0003955	1.246×10^{-4}
☐	oxidoreductase activity, acting on NAD(P)H, quinone o...	GO:0016655	4.157×10^{-4}
☐	active transmembrane transporter activity	GO:0022804	4.607×10^{-4}
☐	oxidoreductase activity, acting on NAD(P)H	GO:0016651	3.625×10^{-3}
☐	oxidoreductase activity, acting on a heme group of do...	GO:0016675	6.340×10^{-3}
☐	cytochrome-c oxidase activity	GO:0004129	6.340×10^{-3}
☑	heparin binding	GO:0008201	7.800×10^{-3}
☐	structural molecule activity	GO:0005198	9.300×10^{-3}
☑	signaling receptor binding	GO:0005102	1.352×10^{-2}
☑	hormone activity	GO:0005179	2.409×10^{-2}
☑	peptidase regulator activity	GO:0061134	3.258×10^{-2}
☐	proton transmembrane transporter activity	GO:0015078	3.921×10^{-2}

$-\log_{10}(p_{adj})$ (0 to ≤16)

Figure S6: Cell Type Proportions on the materal facing side by BMI

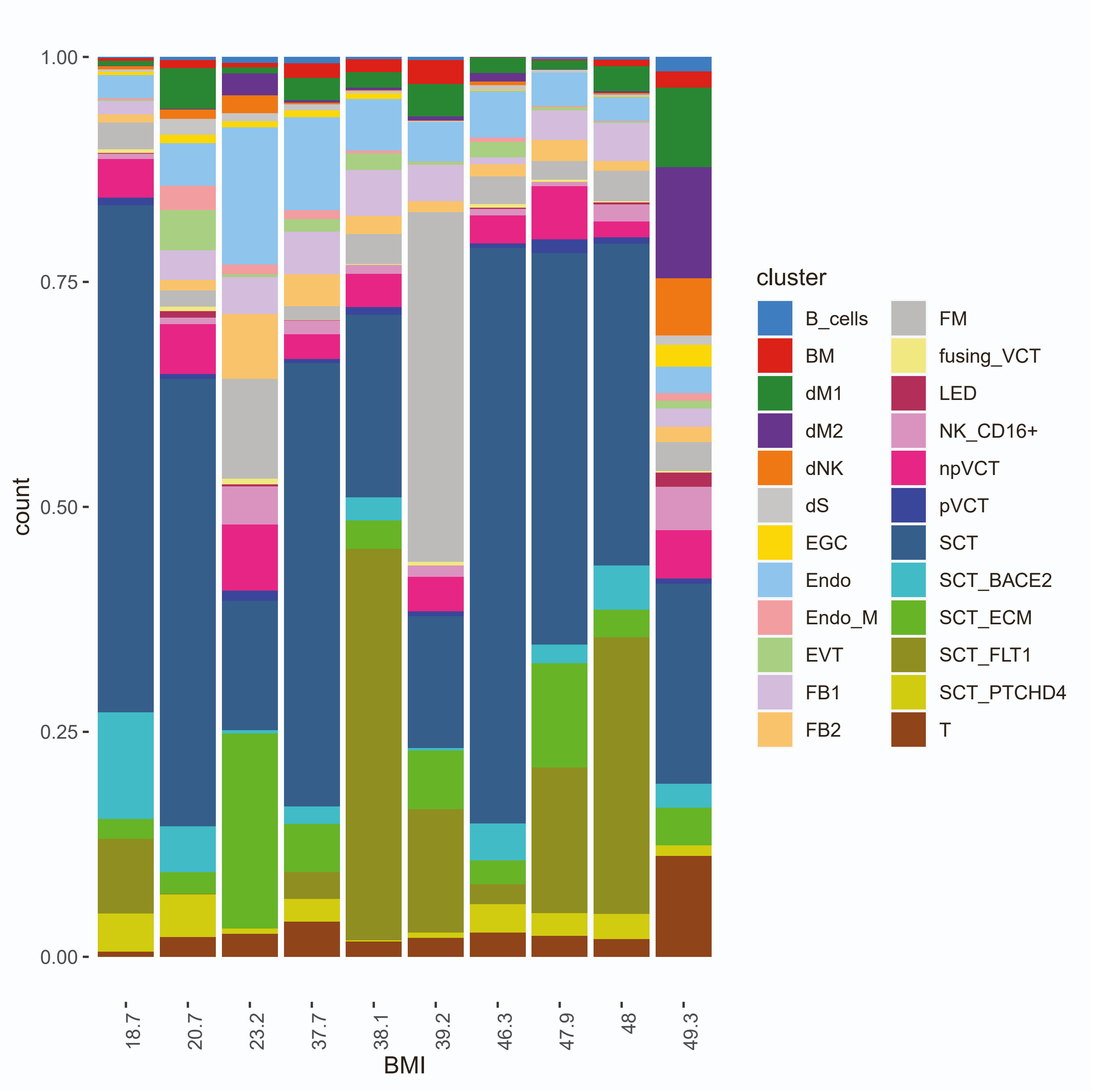

See main Figure 1 legend for abbreviations.

Fig S7: Cell Type Proportions on the fetal facing side by BMI

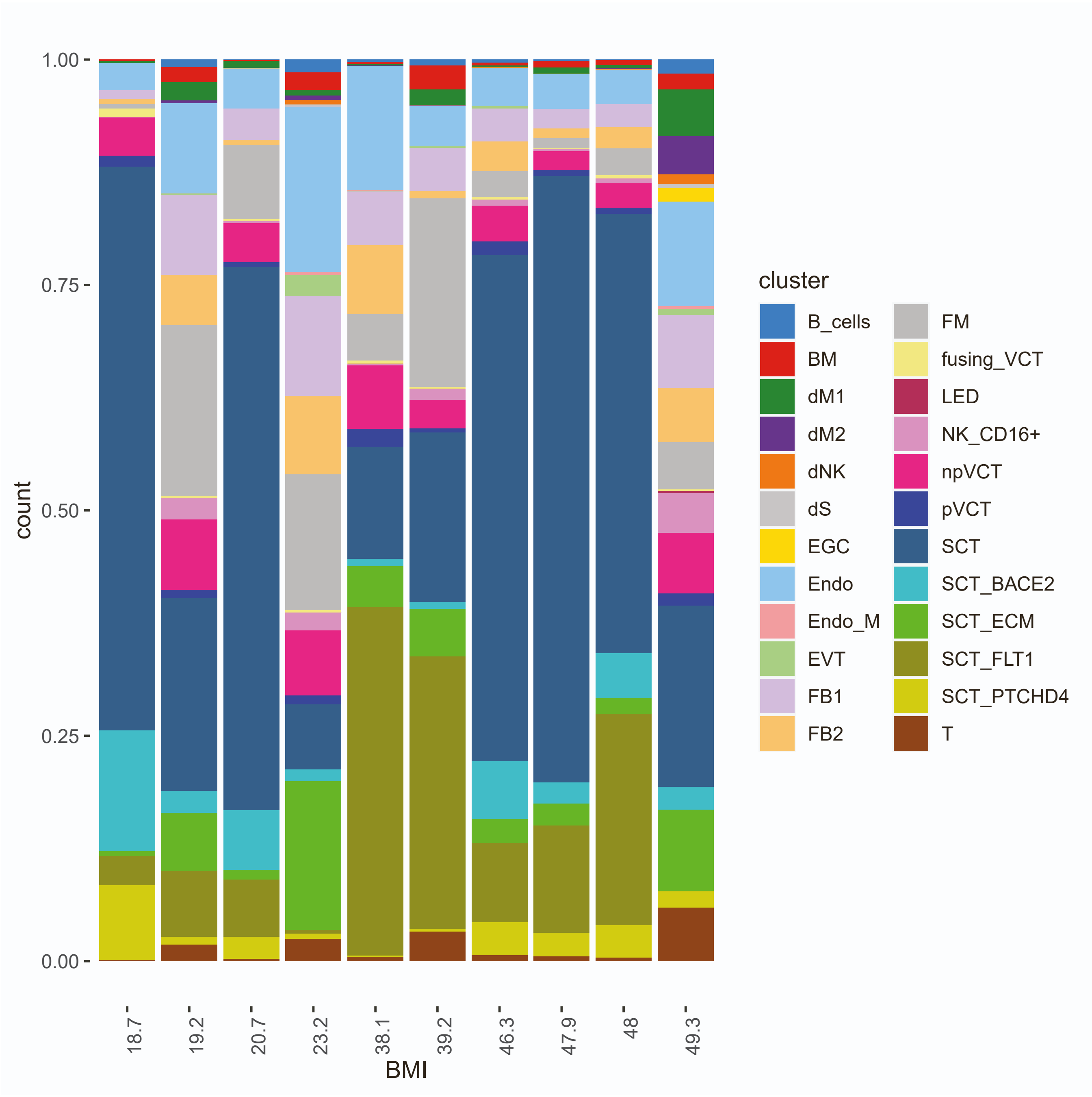

See main Figure 1 legend for abbreviations.

Fig S8: Pathway enrichment based on DEGs from pseudobulked data.

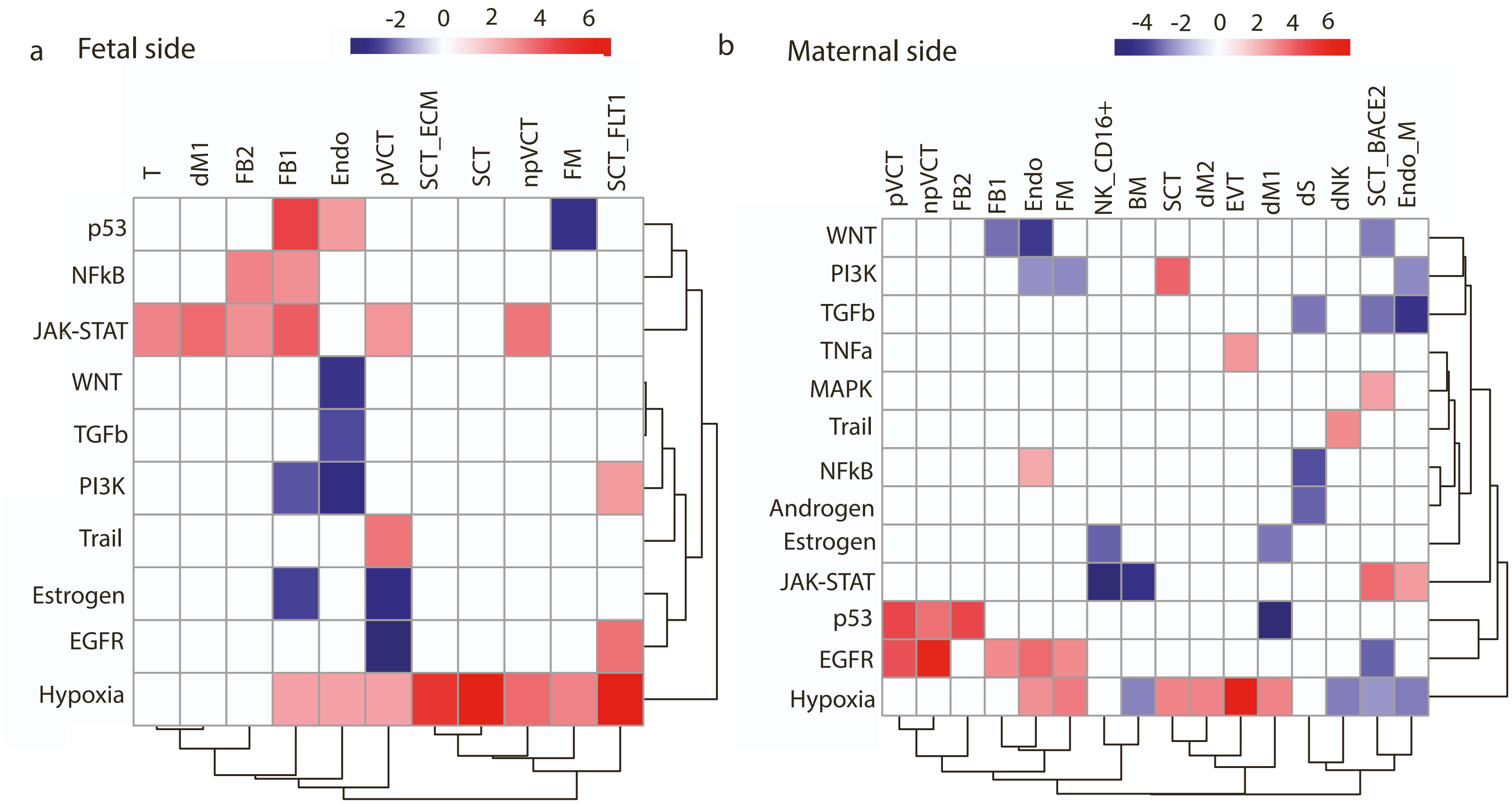

Figure S9: SDQ 5 and ASEBA 5 NBIGS for each nucleus in the term sn-RNA-seq dataset, grouped by cell type

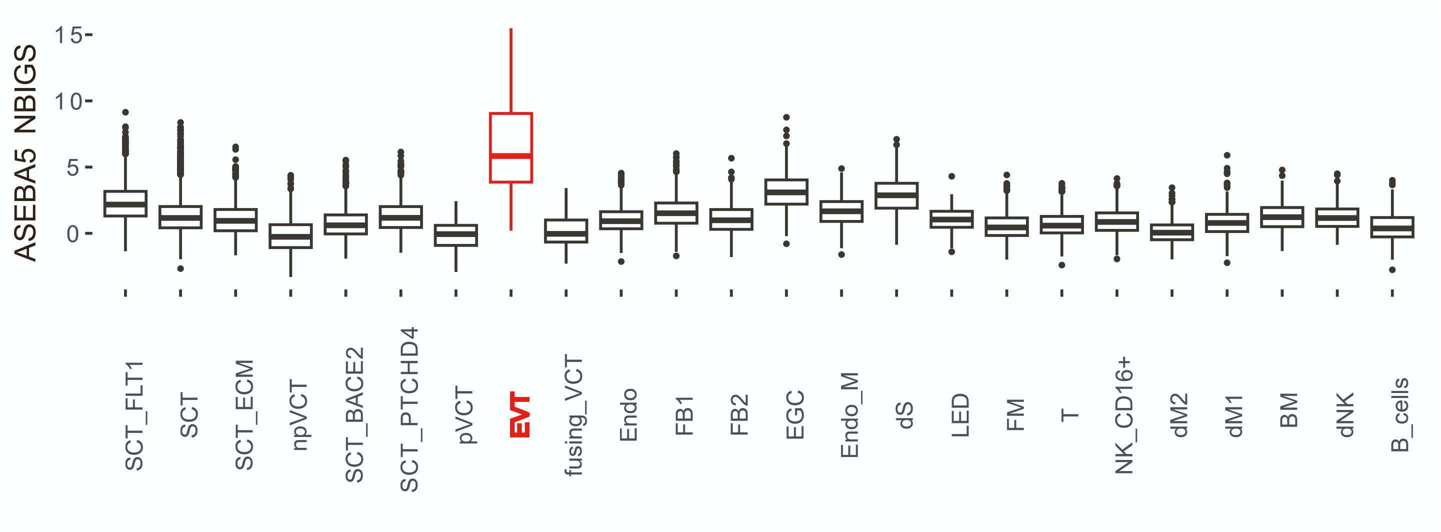

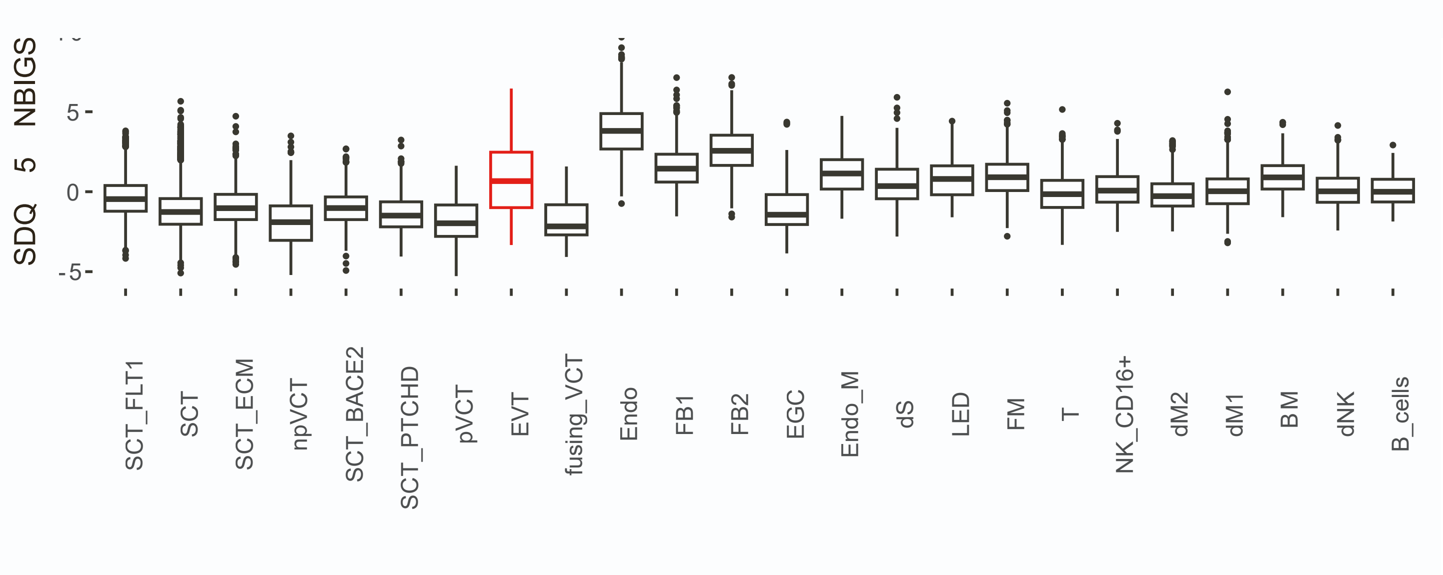

See main Figure 1 legend for abbreviations.

Figure S10: NBIGSs by cell type and BMI category (lean or maternal obesity)

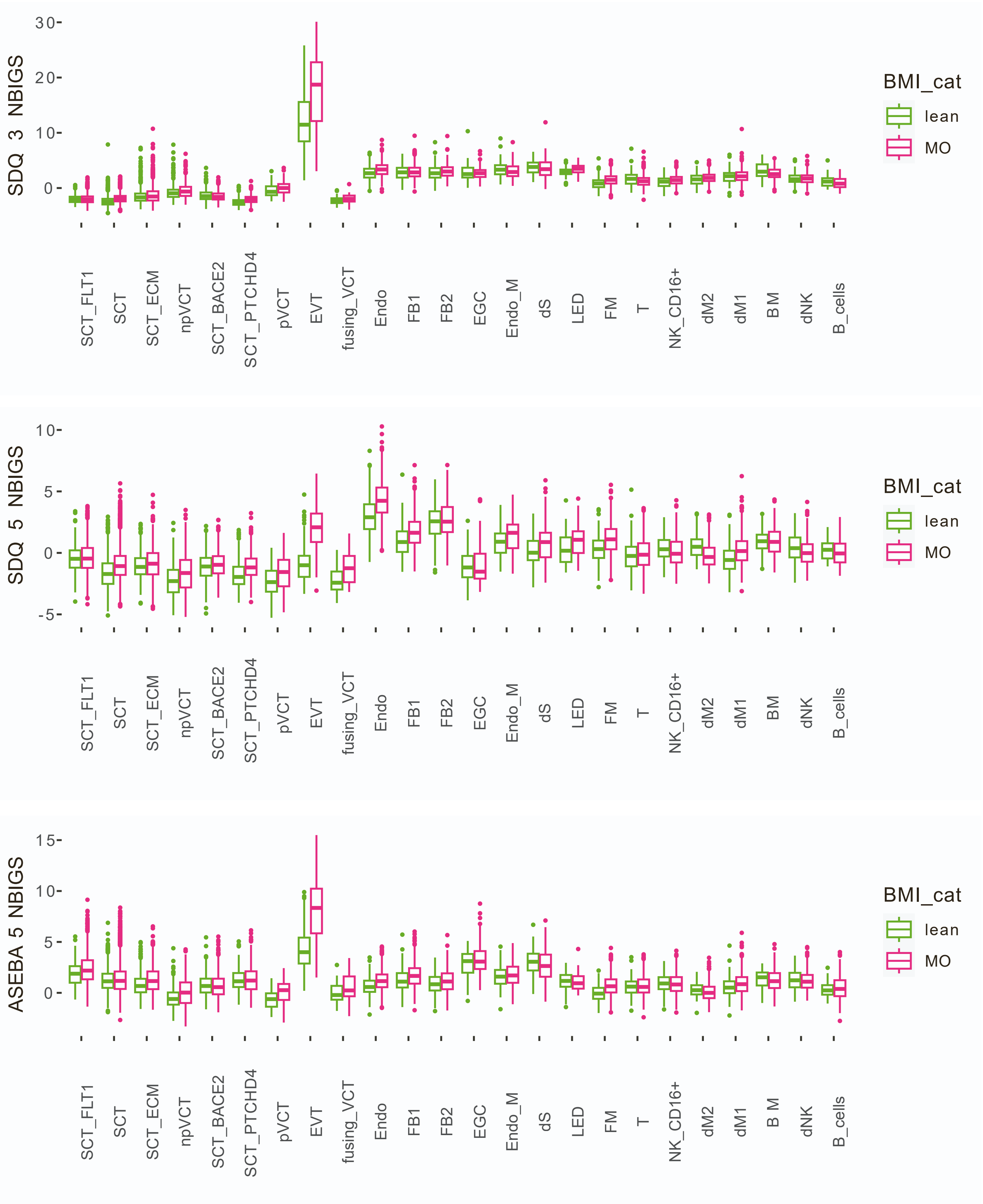

See main Figure 1 legend for abbreviations.

Figure S11: NBIGSs by cell type and fetal sex

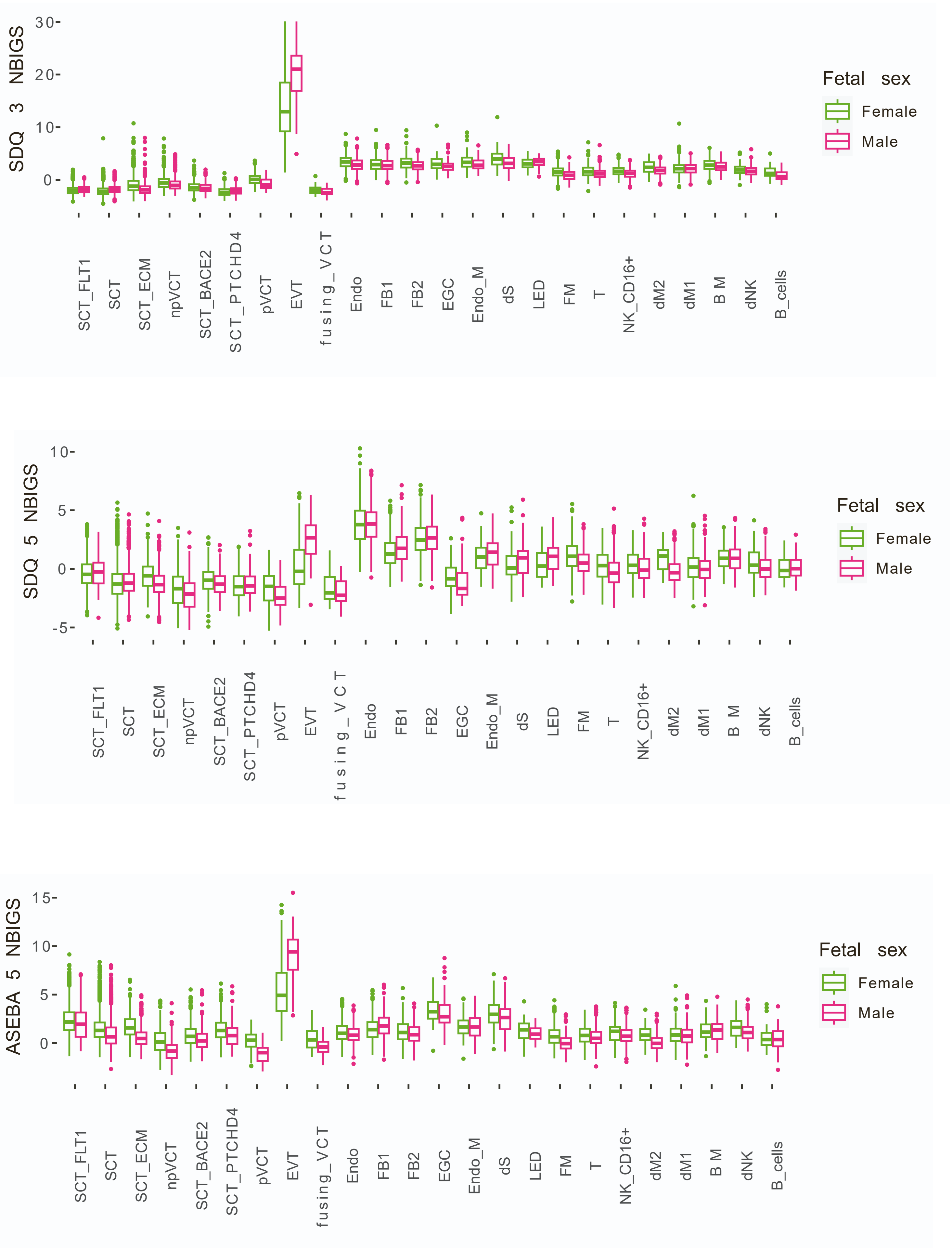

See main Figure 1 legend for abbreviations.

Figure S12: SDQ 5 and ASEBA 5 NBIGS for each cell the second trimester sc-RNA-seq dataset, grouped by cell type

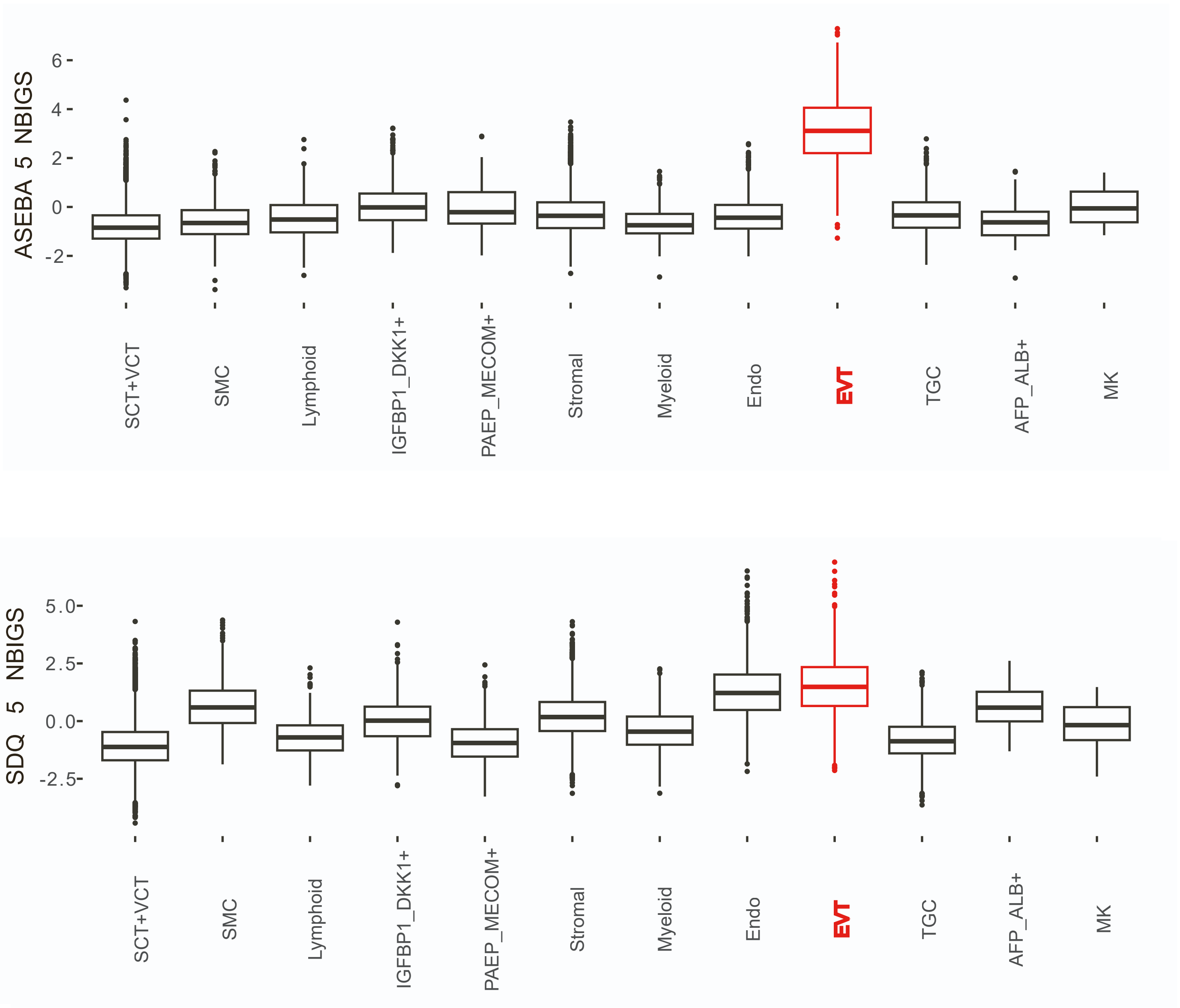

See main Figure 5 legend for abbreviations.

Table S1. Demographic charcteristics of placenta used in the single-nucleus cohort of data generated here.
VI= Vaginal Induction VS: Vaginal Spontaenous CS: Cesarean section

Age at enrollment	Race	Infant sex	BMI	Delivery Mode	Included on maternal side	Included on fetal side
30	White	Female	18.7	VI	yes	yes
25	Black	Female	20.7	VS	yes	yes
27	Black	Male	23.2	CS	yes	yes
19	Black	Male	19.2	CS	no	yes
29	White	Female	38.1	VI	yes	yes
22	Black	Female	39.2	VS	yes	yes
26	Black	Female	46.3	VS	yes	yes
20	Black	Male	37.7	VS	yes	no
30	Black	Male	49.3	CS	yes	yes
33	White	Female	48.0	VI	yes	yes
26	Black	Female	47.9	VS	yes	yes

Table S2. Enrichment of hypoxia associated genes among DEGs by maternal BMI in each cell type, and each placental face based on the NEBULA results.

Pathway	score	p_value	FDR	Cell Type
Hypoxia	5.26	1.48E-07	2.08E-06	ms EVT
Hypoxia	-2.49	1.27E-02	9.37E-02	ms SCT_PTCHD4
Hypoxia	-2.49	1.27E-02	9.37E-02	fs SCT_PTCHD4
Hypoxia	2.46	1.39E-02	9.72E-02	ms dM1
Hypoxia	2.46	1.39E-02	9.72E-02	fs dM1
Hypoxia	2.22	2.66E-02	9.33E-02	ms Endo
Hypoxia	2.22	2.66E-02	9.33E-02	fs Endo
Hypoxia	2.05	3.99E-02	0.48	ms dM2
Hypoxia	-1.61	0.11	0.75	ms dNK
Hypoxia	-1.53	0.13	0.55	ms fusing_VCT
Hypoxia	-1.53	0.13	0.55	fs fusing_VCT
Hypoxia	1.18	0.24	0.83	ms SCT
Hypoxia	1.18	0.24	0.83	fs SCT
Hypoxia	0.96	0.34	0.68	ms pVCT
Hypoxia	0.96	0.34	0.68	fs pVCT
Hypoxia	-0.91	0.36	0.48	ms T
Hypoxia	-0.91	0.36	0.48	fs T
Hypoxia	0.85	0.40	0.62	ms B_cells
Hypoxia	0.83	0.40	0.91	ms npVCT
Hypoxia	0.83	0.40	0.91	fs npVCT
Hypoxia	0.70	0.48	0.85	ms FM
Hypoxia	0.70	0.48	0.85	fs FM
Hypoxia	-0.64	0.52	0.85	ms FB2
Hypoxia	-0.64	0.52	0.85	fs FB2
Hypoxia	-0.63	0.53	0.68	ms SCT_BACE2
Hypoxia	-0.63	0.53	0.68	fs SCT_BACE2
Hypoxia	-0.49	0.63	0.67	ms NK_CD16+
Hypoxia	-0.49	0.63	0.67	fs NK_CD16+
Hypoxia	-0.45	0.65	0.70	ms Blood_macrophage
Hypoxia	-0.45	0.65	0.70	fs Blood_macrophage
Hypoxia	0.43	0.67	0.71	ms dS
Hypoxia	0.43	0.67	0.71	fs dS
Hypoxia	-0.33	0.74	0.80	ms Endo_M
Hypoxia	0.16	0.87	0.87	ms SCT_ECM
Hypoxia	0.16	0.87	0.87	fs SCT_ECM
Hypoxia	0.12	0.91	0.91	ms SCT_FLT1
Hypoxia	0.12	0.91	0.91	fs SCT_FLT1
Hypoxia	-0.11	0.91	0.94	ms FB1
Hypoxia	-0.11	0.91	0.94	fs FB1

Table S3 Test statistics of hypoxia associated genes in the four cell types whose DEGs by BMI had the strongest enrichment for hypoxia response genes.

	ms EVT	fs SCT	fs SCT_FLT1	fs SCT_ECM
ARID3A	0.91	0.45	1.12	-0.10
PFKFB4	1.89	0.67	1.83	0.50
OBSL1	-1.03	-0.26	-0.02	-0.10
TMSB10	-1.48	-0.23	16.04	0.01
PLOD1	1.63	-0.78	NA	-0.13
KDM3A	1.33	-0.34	0.26	-0.54
FLNB	0.49	0.52	0.83	0.32
KDM4B	0.53	0.33	0.73	0.12
MOB3A	0.85	0.31	0.87	0.13
NDRG1	1.54	-0.08	0.99	-0.26
PNRC1	4.67	0.67	2.40	-0.11
FOSL2	0.58	0.61	1.82	0.29
GBE1	0.46	-0.63	0.04	-0.21
SERGEF	0.17	-0.82	-0.18	-1.22
FASTKD2	10.54	-1.12	-0.19	-0.58
CEP41	1.19	-0.89	0.14	0.85
BNIP3L	0.34	-0.23	0.55	-0.05
DPAGT1	5.85	-1.58	-0.05	-0.54
ZMYM2	-0.25	-0.36	0.00	-0.13
PIGA	0.29	-0.49	0.30	-0.79
PKM	-0.03	0.03	1.18	-0.23
SERTAD2	-0.75	0.15	1.84	-0.37
ATP5F1A	-1.38	0.10	1.63	-0.35
KLF7	5.05	-0.37	-2.11	0.81
TPI1	0.48	0.05	1.20	-0.24
MIF	-7.36	-0.55	1.40	-0.78
CRKL	0.24	0.23	1.16	-0.31
TBC1D9B	0.58	0.04	0.86	-0.22
SLC5A6	-1.48	-0.29	-0.31	-0.82
SLC35F6	10.50	-0.24	1.75	-2.02
VCP	-1.31	-0.47	0.03	-0.75
ZNF292	-0.75	-0.34	-0.14	-0.37
BTG1	-0.95	0.05	0.78	-0.86
NUP58	-0.45	-0.53	0.62	-0.63
FEM1C	-0.96	-0.52	0.08	-1.33
ATP5F1B	-4.55	-0.09	1.30	-1.02
TENT4A	0.06	-0.36	-0.16	-0.79
TMEM9B	-5.91	-0.21	0.91	-1.11

NAs come from genes that were not included in the differential expression testing because their expression was too low (<10 counts total across all samples).

Table S4. Demographic charcteristics of placental samples from Gen3G used in this study

	samples form maternal facing side	samples from fetal facing side
N	262	83
Of European descent	98%	99%
Maternal Age	28.7 [25.6-31.4]	28.7 [25.1-31.2]
Fetal Sex	47% female	46% female
Delivery Mode	96% vaginal	95% vaginal
Apgar 5 minutes	9 [9-10]	9 [9-10]
Cord Blood pH	7.26 [7.20-7.30]	7.26 [7.20-7.31]
Hemoglobin	155 [145-168]	154 [145-164]
Birthweight	3465 [3225-3725]	3470 [3225-3705]

Values represent median [interquartile range] or percentage.

Table S5. Results of Spearman correlation between hypoxia gene score and offspring measures.

category	test	side	cor	p
Acute	APGAR at 5 min	fs	-0.344	3.09E-04
Acute	APGAR at 5 min	ms	-0.123	4.70E-02
Acute	Cord Blood pH	fs	-0.413	2.87E-05
Acute	Cord Blood pH	ms	-0.247	8.10E-05
Acute	Hemoglobin	fs	0.318	8.54E-04
Acute	Hemoglobin	ms	0.140	2.42E-02
Growth	Birthweight	fs	-0.197	4.24E-02
Growth	Birthweight	ms	0.009	8.88E-01
Growth	BMI at age 3	fs	0.045	7.10E-01
Growth	BMI at age 3	ms	0.185	2.57E-02
Growth	BMI at age 5	fs	0.039	7.25E-01
Growth	BMI at age 5	ms	0.029	7.01E-01
Neurobehavior	ASEBA at age 5	fs	0.028	8.10E-01
Neurobehavior	ASEBA at age 5	ms	0.167	2.74E-02
Neurobehavior	SDQ at age 3	fs	0.174	1.53E-01
Neurobehavior	SDQ at age 3	ms	0.256	1.50E-03
Neurobehavior	SDQ at age 5	fs	0.010	9.30E-01
Neurobehavior	SDQ at age 5	ms	0.181	1.38E-02

Table S6: Correlations between neurobehavioral gene scores (NBIGSs) and hypoxia gene score (HGS) for each cell type in the term placenta sn-RNA-seq dataset

Cell Type	SDQ 3 cor	SDQ 3 p	SDQ 5 cor	SDQ 5 p	ASEBA 5 cor	ASEBA 5 p
EVT	0.537	<2.2e-16	0.550	<2.2e-16	0.462	<2.2e-16
Endo_M	0.392	1.06E-09	0.357	3.52E-08	0.067	3.10E-01
dS	0.388	2.28E-08	0.417	1.51E-09	-0.021	7.69E-01
Endo	0.314	3.22E-39	0.003	8.94E-01	0.048	5.11E-02
EGC	0.251	2.26E-04	0.212	1.97E-03	0.074	2.84E-01
FM	0.221	9.04E-19	0.124	7.37E-07	0.006	8.22E-01
FB1	0.214	1.60E-11	-0.002	9.57E-01	-0.105	9.68E-04
dNK	0.194	1.50E-04	0.062	2.31E-01	-0.092	7.32E-02
dM2	0.172	1.61E-05	-0.052	1.97E-01	-0.106	7.94E-03
FB2	0.137	4.77E-04	-0.033	4.01E-01	-0.039	3.25E-01
BM	0.128	2.46E-02	0.031	5.93E-01	-0.027	6.35E-01
SCT_BACE2	0.110	3.03E-04	0.084	6.13E-03	0.072	1.82E-02
pVCT	0.099	1.48E-01	0.162	1.67E-02	-0.026	7.05E-01
dM1	0.094	4.39E-03	0.124	1.72E-04	-0.034	3.01E-01
LED	0.088	3.60E-01	0.188	5.03E-02	0.023	8.14E-01
T	0.076	1.48E-02	-0.059	5.91E-02	-0.208	2.35E-11
NK_CD16+	0.070	1.03E-01	0.001	9.84E-01	-0.206	1.51E-06
npVCT	0.028	3.17E-01	0.026	3.63E-01	-0.120	2.35E-05
B_cells	-0.015	8.51E-01	0.257	1.23E-03	-0.015	8.52E-01
fusing_VCT	-0.032	7.76E-01	-0.131	2.39E-01	-0.205	6.54E-02
SCT_ECM	-0.032	1.94E-01	0.215	1.54E-18	0.008	7.51E-01
SCT_FLT1	-0.117	1.84E-12	0.277	7.72E-64	0.143	7.09E-18
SCT	-0.129	2.28E-41	0.180	6.41E-79	-0.018	6.29E-02
SCT_PTCHD4	-0.213	3.50E-08	0.177	5.30E-06	-0.062	1.12E-01

Table S7: Correlations between NBIGSs and HGS for each cell type in the second trimester placenta sc-RNA-seq dataset

Cell Type	SDQ 3 cor	SDQ 3 p	SDQ 5 cor	SDQ 5 p	ASEBA 5 cor	ASEBA 5 p
EVT	0.285	3.60E-30	0.243	4.07E-22	0.175	5.72E-12
MK	0.285	1.27E-01	0.152	4.20E-01	-0.067	7.24E-01
IGFBP1_DKK1+	0.193	2.61E-07	0.076	4.49E-02	0.069	6.87E-02
Stromal	0.190	9.77E-69	0.079	5.04E-13	-0.020	6.69E-02
PAEP_MECOM+	0.168	4.30E-03	0.011	8.54E-01	0.124	3.58E-02
SCT+VCT	0.154	3.84E-78	0.172	2.24E-97	0.067	4.66E-16
Endo	0.145	2.65E-08	0.061	1.98E-02	0.015	5.79E-01
SMC	0.127	1.66E-04	0.081	1.63E-02	-0.048	1.57E-01
Lymphoid	0.104	2.53E-02	0.076	1.01E-01	0.037	4.28E-01
Myeloid	0.043	4.30E-01	0.043	4.26E-01	0.065	2.33E-01
TGC	0.033	2.62E-01	0.115	1.01E-04	0.061	3.85E-02
AFP_ALB+	-0.028	7.98E-01	0.036	7.37E-01	0.013	9.03E-01
